# Random cellulose acetate nanofibers: a breakthrough for cultivated meat production

**DOI:** 10.3389/fnut.2023.1297926

**Published:** 2024-01-05

**Authors:** Ana Elisa Antunes dos Santos, Jorge Luís Guadalupe, Juliano Douglas Silva Albergaria, Itallo Augusto Almeida, Amanda Maria Siqueira Moreira, Aline Gonçalves Lio Copola, Isabella Paula de Araújo, Ana Maria de Paula, Bernardo Ruegger Almeida Neves, João Paulo Ferreira Santos, Aline Bruna da Silva, Erika Cristina Jorge, Luciana de Oliveira Andrade

**Affiliations:** ^1^Department of Morphology, Institute of Biological Science, Federal University of Minas Gerais, Belo Horizonte, Brazil; ^2^Laboratory of Biomaterials, Department of Materials Engineering, Federal Center for Technological Education of Minas Gerais (CEFET-MG), Belo Horizonte, Brazil; ^3^Department of Physics, Institute of Exact Sciences, Federal University of Minas Gerais, Belo Horizonte, Minas Gerais, Brazil

**Keywords:** cellulose acetate, nanofiber, scaffold, muscle tissue engineering, cultivated meat

## Abstract

Overcoming the challenge of creating thick, tissue-resembling muscle constructs is paramount in the field of cultivated meat production. This study investigates the remarkable potential of random cellulose acetate nanofibers (CAN) as a transformative scaffold for muscle tissue engineering (MTE), specifically in the context of cultivated meat applications. Through a comparative analysis between random and aligned CAN, utilizing C2C12 and H9c2 myoblasts, we unveil the unparalleled capabilities of random CAN in facilitating muscle differentiation, independent of differentiation media, by exploiting the YAP/TAZ-related mechanotransduction pathway. In addition, we have successfully developed a novel process for stacking cell-loaded CAN sheets, enabling the production of a three-dimensional meat product. C2C12 and H9c2 loaded CAN sheets were stacked (up to four layers) to form a ~300–400 μm thick tissue 2 cm in length, organized in a mesh of uniaxial aligned cells. To further demonstrate the effectiveness of this methodology for cultivated meat purposes, we have generated thick and viable constructs using chicken muscle satellite cells (cSCs) and random CAN. This groundbreaking discovery offers a cost-effective and biomimetic solution for cultivating and differentiating muscle cells, forging a crucial link between tissue engineering and the pursuit of sustainable and affordable cultivated meat production.

## 1 Introduction

Muscle tissue engineering (MTE) has evolved to encompass a wide range of applications, displaying remarkable versatility. Initially, the focus of MTE was on developing constructs for biomedical purposes, aiming to restore damaged skeletal and cardiac muscles (1, 2). Also, the development of 3D constructs that better resemble *in situ* tissue and replicate key aspects of human physiology, has been key to exempting the use of animal models in research (3–5). Notably, the pioneering work of Mark Post in 2013 brought forth a groundbreaking application of skeletal MTE directed to the food industry (6). This breakthrough led to the emergence of cultivated meat, a technology that circumvents the necessity for traditional animal farming and slaughter. The concept of cultivated meat has since garnered substantial attention from the scientific community and the market, highlighting the need for continuous optimization of MTE constructs (7–9). Being able to produce a whole cut of cultivated meat that faithfully replicates the diverse arrangement of cells present in authentic animal muscle tissue, within a 3D structure, still stands as a significant challenge in the realm of cultivated meat technology (10).

In order to be able to recreate tissue 3D structure, which resembles the *in vivo* tissue, the use of scaffolds is paramount, being one of the most explored fields in tissue engineering (11, 12). The most used are porous, hydrogel, and fiber scaffolds, as well as additive methods, like 3D bioprinting, using hydrogel-based bio-inks. Each scaffold type brings unique attributes and applications, contributing to the advancement of tissue engineering techniques (13).

In cultivated meat production, the primary purpose of the scaffold is to support the development of muscles, fat, and connective tissue. However, to create a structured meat product resembling a whole cut of meat, like a filet or a steak, a suitable scaffolding approach is necessary. This approach should facilitate cell proliferation, differentiation into essential cell types, and spatial arrangement to achieve the familiar appearance and texture of popular meat cuts. Electrospinning has garnered significant interest as a versatile technique capable of producing fibrous structures with fiber diameters ranging from a few nanometers to microns (14, 15). The fibers obtained by electrospinning offer many advantages such as a high surface-area-to-volume ratio and the ability to modulate both pore size, facilitating the support of high cell density, and fiber arrangement (random or aligned). Additionally, the versatility of using different biomaterials.^1^ These features make electrospun nanofibers a promising scaffold for applications in tissue engineering of cultivated structured meat. Although most investigations on nanofiber scaffolding for MTE have focused on the biomedical field, MacQueen and coworkers, as well as Santos and coworkers have demonstrated their potential for cultivated meat applications (16, 17).

Another important feature of a scaffold for MTE is not only its permissibility for cellular colonization, but also its potential for cell differentiation. From the physical cues capable of driving muscle cell differentiation, topography, mainly micropatterning alignment, is usually the main designed feature. Thus, aligned nanofiber scaffolds are typically preferred in MTE studies due to their ability to guide cell alignment through topographical cues (18–20). However, the core purpose of a scaffold is to mimic the native tissue’s extracellular matrix (ECM). In this sense, the muscle endomysium (ECM involving each muscle fiber) is mainly composed of a random mesh of collagen fibrils (21, 22) and mimicking this fiber disposition might be important for cultivated meat taste and texture.

It is also well-established that the mechanical properties of the scaffold, on which the cells are placed, can have an impact as significant as the addition of known tissue differentiation molecules (23). Specifically, the elasticity of the polymer serves as a crucial cue that influences various mechanosensing processes in the cells (23, 24). However, until now, little knowledge has been gathered on how to leverage the scaffold’s elasticity to prompt myogenic differentiation in a cultured meat bioprocess scenario. Despite that, some studies have pointed out that substrate’s stiffness is a key regulator of muscle cells gene transcription and muscle-like stiffness stimulates myogenesis (25, 26). Furthermore, for cultivated meat applications, substrate stiffness could play a significant role in enhancing the sensory attributes such as taste, mouthfeel, and texture (8), underscoring its investigation and characterization.

Finally, it is important to highlight that the success of MTE technology will lay upon the ability of creating a thick construct that is also easily scaled-up. However, the biofabrication of muscle tissues faces several hurdles when recreating a thick structure. Without the presence of capillary perfusion, the diffusion of nutrients and respiratory gases is limited and will depend on structures with larger pores, which may compromise tissue organization (27, 28). Nonetheless, the delivery of nutrients in thicknesses of several millimeters must be achieved. Overcoming this hurdle and enabling cells to survive within the tissue remains among the most crucial challenges in the field of cultivated meat.

Cellulose acetate is a soluble, processable, and biodegradable polymer (29) that has been widely produced through electrospinning for tissue engineering purposes (30). Here, we evaluate the potential of random and aligned cellulose acetate nanofibers (CAN) as scaffolds for MTE applications, using mouse C2C12 and rat H9c2 muscle cell lines. The latter, unlike C2C12, can differentiate into both skeletal and cardiac muscle cells, depending on growth conditions. While confluence will direct H9c2 differentiation toward skeletal muscle (31), exposure to retinoic acid shifts its differentiation into cardiac muscle (32), making it an interesting model also for biomedical uses.

Our study revealed that random CAN were able to induce myoblast differentiation even in growth medium conditions, without any external chemical stimuli. This substrate-induced differentiation involved the mechanotransduction Hippo signaling pathway, coordinated by YAP/TAZ proteins. Upon this characterization, we addressed the challenge of biofabricating a thick tissue for cultured meat purposes, by using a simple novel strategy, in which individual layers of porous random CAN were stacked during the culture of immortalized muscle cells. This new methodology not only supported cell viability throughout the entire construct, but also provided a favorable environment for cell alignment. Subsequently, we extended this novel methodology to primary chicken myoblasts (cSCs), demonstrating its effectiveness for both immortalized and primary myoblasts. These findings are groundbreaking as they introduce a substrate with architecture resembling the *in vivo* muscle extracellular matrix (ECM), offering several advantages: (I) induction of muscle differentiation without the reliance on costly alternative media formulations for cultivated meat applications; (II) facilitation of cell alignment, mimicking natural muscle architecture without additional topographical cues; and (III) support for multi-layered culture, maintaining cellular viability even within the inner sections of the construct. Employing this technique, we successfully assembled chicken muscle sheets into a cohesive and structured meat product for cultivated meat production purposes.

## 2 Materials and methods

### 2.1 Material preparation

Cellulose acetate (CA) in powder form, with Mn ~30,000 g/mol, density of 1.3 g/mL, and 40% degree of substitution was purchased from Sigma-Aldrich (São Paulo). The solvent used for electrospinning was acetone and N, N-dimethylformamide (DMF), all from Labsynth (Diadema, Brazil). For the cellulose acetate solution, cellulose acetate was dissolved in acetone-dimethylformamide (3:1 v/v) to obtain a 12% (w/v) solution, as previously described (33). The cellulose acetate nanofibers (CA) were obtained by electrospinning, employing the following parameters: voltage 16 kV, collecting distance 14 cm, collector rotation at 400 rpm, and solution gravity-fed at room temperature. Regarding the rotation of the collector, it presented two parameters, 400 rpm for obtaining a film formed by nanofibers deposited with different orientations (random) and 1,500 rpm for deposition of nanofibers oriented in parallel (aligned). Subsequently, the nanofibrous membranes were dried in a vacuum chamber for 3 days to remove the remaining solvents.

### 2.2 Material characterization

The membrane samples were characterized by SEM and AFM for surface morphology and roughness analysis. For SEM observation, the samples were sprayed with a layer of gold for 20 min using a sputter coater (Cressington 108 model; Cressington Scientific Instruments, Watford, England), and then examined utilizing a Quanta 200 FEG (FEI, Hillsboro, USA) scanning electron microscope, using medium vacuum (60 Pa) and auto focus on an accelerating voltage of 5 kV. Average fiber diameter size distribution and thickness were obtained upon analysis of scanning electron microscopy (SEM) images, using ImageJ software. From three SEM images of each sample, 200 fibers were randomly selected and their diameter measured manually using the Line tool. The surface roughness of the CAN were analyzed using an Asylum Research MFP-3D-SA Atomic Force Microscope (AFM), using a contactless module with a force of 1 nN in the 30×30 μm area. Average pore areas were obtained upon analysis of AFM images, using ImageJ software. From three AFM images of each sample, 10 pore were randomly selected and their area measured manually using the freehand selection tool. For scaffold thickness, nine SEM images of each sample were selected, and three sample thickness were randomly measured. The thickness were measured manually using the ImageJ software line tool.

### 2.3 Cell culture

Immortalized mouse myoblasts (C2C12) and rat cardiomyoblasts (H9c2) were maintained in tissue culture flasks in growth medium (GM) [GM: DMEM-high glucose (Gibco), supplemented with 10% bovine fetal serum (Gibco) and 1% pen-strep (Gibco)]. For 3D culture, cells were seeded onto the CAN scaffolds. Before cell seeding, scaffolds with 120–140 μm thickness were cut into 16 mm diameter discs and fixed in the well of a 24-well plate and sterilized using gamma irradiation. The materials were irradiated at room temperature with a standard dose of 10 kGy. Cobalt-60 (60Co) gamma-ray source was used. Gamma irradiation sterilization was carried out at Gamma Irradiation Laboratory installed at the Nuclear Technology Development Centre (CDTN), Belo Horizonte, Brazil. Both CAN scaffolds, random and aligned, were equilibrated using 200 μL of growth medium (GM) for 15 min before cell seeding. C2C12 and H9c2 cells were seeded in triplicates always below the 8th passage. Monolayer cultures, plated over glass coverslips, were used as controls. For both monolayer and CAN, cells were seeded at a density of 1×10^5^ or 5×10^5^ cells/well in 24-well plates. 2 h of incubation at 37°C and 5% CO_2_, the volume of GM was completed to 500 μL/well and the growth medium was replaced every 2 days. For differentiation induction of C2C12, after 3 days of culture in GM the medium was changed to differentiation medium (DM) [DM: DMEM-high glucose (Gibco), supplemented with 2% horse serum (Gibco) and 1% pen-strep (Gibco). For H9c2, after 3 days of culture, the GM was changed for differentiation medium (DM) [DM: DMEM-high glucose (Gibco), supplemented with 1% bovine serum (Gibco), 1% pen-strep (Gibco) and 10 nM of retinoic acid]. The DM was replaced every 2 days. Chicken satellite cells (cSCs) were successfully obtained from biopsies of pectoralis and iliotibialis muscle of avian embryos at 10 and 15 days (E10 and E15). In brief, cells were mechanically and chemically dissociated by digestion with collagenase I (Gibco), followed by digestion with 0.25% trypsin, both at 37°C. cSCs were enriched using the selective adhesion method, which consists of cell incubation for 1 h at 37°C and 5% CO_2_ in growth medium [GM: DMEM-high glucose (Gibco), supplemented with 20% bovine fetal serum (Gibco) and 1% anti-anti (Gibco)], followed by definitive plating of the supernatant. The skeletal muscle phenotype of the cSCs after up to 3 enrichment cycles was confirmed by immunofluorescence using myogenic markers.

### 2.4 Cell counting

Cells were seeded onto the scaffolds and as a monolayer at a density of 1×10^5^ cells/well in GM. After 24 h, supernatants were carefully collected and transferred to a new tube. The well was carefully washed with PBS, which was also transferred to the same tube containing the supernatant. Tubes containing well supernatant and washed PBS from each individual well were centrifuged at 400× *g* for 7 min, and each pellet was resuspended in 50 μL of fresh GM. Cells were counted using a Neubauer chamber. For cell seeding efficiency with and without medium supplemented with fetal bovine serum (FBS), 1×10^5^ C2C12 cells and 5×10^5^ H9c2 cells were plated on the nanofiber (random and aligned) and on the monolayer, in triplicate for each group. After 24 h, the medium was removed and the supernatant cells were counted in Neubauer’s chambers. Seeding efficiency was then calculated as:


$$\mathrm{Seeding}\;\mathrm{efficiency}\;\left(\%\right)=\frac{\mathrm{Initialcells}-\mathrm{Supernatantcells}}{\mathrm{Initialcells}}\times 100$$

### 2.5 Cell viability and proliferation

Cell viability was assessed using MTT (3-(4,5-dimethylthiazol-2-yl)-2,5-diphenyl tetrazolium bromide) (Invitrogen) and Live/dead staining (Invitrogen). For MTT assay, C2C12 and H9c2 cells were seeded onto the scaffolds or as a monolayer at a density of 1×10^5^ cells/well in a 24-well plate in GM. After 1, 2 and 3 days, GM was replaced with the MTT solution, and the samples were incubated for 2 h at 37°C and 5% CO_2_. Formazan crystals were then dissolved in 1 mL/well of isopropanol. Next, the solution was then transferred to a 96-well plate in triplicate and absorbance were measured at 595 nm using a microplate reader (Multiskan FC, Thermo Scientific, USA). For live/dead staining, C2C12 and H9c2 were stained with 2 μM calcein-AM and 4 μM ethidium homodimer-1 (Invitrogen) in PBS at 37°C for 30 min, followed by rinsing with PBS 1x and fluorescence imaging by fluorescence microscopy (Zeiss Axiovert.A1) with 10x objective lens. Alternatively, another proliferation assay was performed using DAPI-labeled nuclei counting. Six pictures of six fields were taken on a Zeiss Axiovert.A1 fluorescence microscope with a 10x objective lens of each of the three replicates. Nuclei were quantified using ImageJ software. For this, czi images were converted to 8-bit images, the threshold was set manually and the path process-binary-watershed was executed, after which the analyze-analyze particles-size (3000-infinity)-summarize results-display results path was performed.

### 2.6 SEM and fluorescence morphological assays

The morphology of the cells along the time in the different substrates was first analyzed by SEM. For this, the samples were fixed with 2.5% (v/v) glutaraldehyde in 0.1 M phosphate buffer for 2 h at RT. The samples were rinsed with phosphate buffer and gradually dehydrated in an alcohol series of increasing concentration (35, 50, 70, 85, 95 and 100% for 15 min/bath), followed by critical point drying, and gold coating. Images were captured using Quanta 200 FEG SEM (FEI, Hillsboro, USA). Cell morphology was also accessed by actin labeling. For this, the scaffolds or coverslips containing the cells were washed twice with PBS and then fixed with formaldehyde 3.7% at RT for 20 min. The samples were then washed with PBS, permeabilized with Triton-X100 0.5% for 15 min at RT, washed again with PBS, and incubated with Alexa Fluor 546 phalloidin 1:100 in PBS for 30 min at RT. Next, the cells were washed with PBS and cell nuclei were stained with DAPI (diluted to 1:1,000 in PBS) for 1 min at RT. Scaffold samples were first put over coverslips before all samples were mounted in glass slides using hydromount and visualized in either a fluorescence microscope Zeiss Axiovert.A1 or a confocal microscope Zeiss LSM 880. Cell elongation was quantitatively measured by nuclear aspect ratio (NAR), which were defined as the ratio between the length of the longest line to the length of the shortest line across the nuclei, using ImageJ software. Six nuclei from five fields from each of two biological replicates were randomly selected. NAR was manually measured using the line tool. Cell alignment was also executed using the angle tool. An arbitrary line pointing the direction of the majority of the cells was drawn in each field and the angle of it with five randomly selected cells was measured. Five fields from two independent experiments were selected. Polar graphs were designed on Origin Pro software.

### 2.7 Cell infiltration

Actin coverslips analyzed in the LSM 880 confocal microscope were picked for quantification of cell infiltration into aCAN and rCAN at 24, 48, and 72 h. For that, a stochastic selection of five fields from each of the three replicates was placed. In each of them, the z-depth analysis, given by the z-stack configuration, was collected. For the infiltration analysis of the stacked construct layers, 6 fields from each layer of two independent stacks were collected.

### 2.8 Gene expression

Gene expression was analyzed using real-time quantitative polymerase chain reaction (RT-qPCR). All cells from the triplicate were then harvested in 1 mL TriReagent (Sigma-Aldrich) and the total RNA was isolated according to the manufacturer’s instructions. Next, 1 μg of each total RNA sample was converted into cDNA, following the instructions in the RevertAid H minus first strand cDNA synthesis kit (Thermo Fischer Scientific). RT-qPCR was performed using a Corbett 3,000 device (Qiagen, Helden, Germany), 0.4–0.8 μM of each primer, 1 μL (diluted 1:10) of each cDNA, and 5 μL of iTaq Universal SYBR Green Supermix (Bio-Rad, Hercules, USA) in a final volume of 10 μL. Reactions were performed as follows: 50°C for 2 min, 95°C for 2 min, followed by 45 cycles of 94°C for 15 s, 60–62°C for 15 s, and 72 ° for 20 s. The dissociation step was performed at the end of the amplification step to identify the specific melting temperature for each primer set. GAPDH was used as a reference gene. Primer’s sequences are represented in Supplementary Table 1. Relative gene expression was determined using REST2009 software (based on the model by Pfaffl et al.) (34, 35). The relative gene expression for each gene was determined by comparing 1-day expression levels in GM in cells cultivated onto scaffolds and in cells cultivated in a monolayer. REST 2009 software was used to determine statistical significance.

### 2.9 Mechanical test

The Young’s modulus (E) of each nanofiber mesh (aCAN and rCAN) was measured using an Asylum Research MFP-3D Atomic Force Microscope (AFM) in force spectroscopy mode. The nanofiber meshes were fixed on glass coverslips with alginate glue and analyzed with a SAASPH-5UM probe (Bruker) with a 10 μm diameter spherical tip. The spring constants of the probes were individually calibrated using the Thermal Tuning method. Single indentations were performed with an applied force of 12 nN. Young’s modulus values were determined by averaging over five unique indentations per nanofiber mesh (aCAN and rCAN). Force curve analysis was performed with the Asylum Research Data Processing software employing the JKR model.

### 2.10 Immunofluorescence

For YAP staining, 1×10^4^ C2C12 cells were seeded in each 24-well plated onto the coverslip, aCAN, and rCAN. 24 h later, they were fixed with PFA 4%, incubated with 0,1% Triton X-100 for 1 h, followed by PBS/BSA 5% for 1 h for blocking. Anti-YAP/TAZ (Santa Cruz Biotechnology, SC-101199; 1:250) was used as the primary antibody and incubated for 1 h at RT. After three washes in PBST (tween 20 0,5%), Alexa-fluor 488 conjugated secondary antibody was added in PBS/BSA 5%. After 1 h, cells were washed with PBS, stained for DAPI, mounted with hydromount, and visualized in Zeiss Axiovert A.1. For Myosin Heavy Chain visualization, cSCs were washed in PBS, fixed with 4% paraformaldehyde for 15 min, blocked in 0,1 Glycine in PBS for 15 min, well washed in PBS, permeabilized and blocked with a solution of 0,5% Goat Serum and 0,2% TritonX, in PBS, during 30 min, and incubated at 4°C, overnight, with primary mouse anti-chicken-myosin heavy-chain antibody (MF20 monoclonal; Hybridoma Bank, diluted 1:100). Cells were then washed in PBS and incubated for 90 min in darkroom, at room temperature, with the secondary antibody Alexa Fluor 555 donkey anti-mouse IgG (Thermo Fisher) diluted 1:500 in PBS. Immunocytochemistry images were obtained by microscopy with the fluorescence capture system (BX41 Olympus microscope with Q-Color 3/Olympus capture system). Image J software was used for image assembly.

### 2.11 Manipulation and stacking process

For stacking, 5×10^5^ H9c2 and C2C12 cells were plated per nanofiber, separately. After plating, cells were maintained in growth medium (DMEM, supplemented with 10% fetal bovine serum and 1% pen-strep) for 2 days. In our first test, the nanofiber mesh (nanofiber and cells) was carefully separated from the 24-well plate using a glass coverslip and placed on top of another nanofiber mesh. After stacking, the cells were kept for two more days in GM. After this process, the cells were fixed and processed for morphological evaluation of cell appearance and organization, through phalloidin labeling and evaluation of the actin cytoskeleton via fluorescence microscopy. Then the stacking was gradually increased to three and four layers. For the 3-layers stacking, after 2 days, two layers of nanofibers were stacked one on top of the other and after another 2 days, the third layer was stacked on top of the other two. For the 3-layer stacking, the cells were analyzed through: 1. Live/Dead assay for viability analysis; 2. Phalloidin labeling and evaluation of the actin cytoskeleton via fluorescence microscopy. For the 4-layers stacking, this process of stacking two nanofibers every 2 days was repeated two times until reaching the 4-layers stacking. After this process, the cells were evaluated through: 1. Live/Dead assay for viability analysis; 2. Phalloidin labeling and evaluation of the actin cytoskeleton via fluorescence microscopy; 3. Histological staining. Throughout the stacking process, cells were incubated in GM at 37°C and 5% CO_2_, and the total well volume was 500 μL. The entire stacking process was done manually, with sterile tweezers and needles.

### 2.12 Histological staining

After 6 days of culture, the stacked construct was fixed in 4% PFA and prepared for histology. Because the inclusion of nanofibers for the occurrence of cuts was difficult due to their high solubility in conventional reagents such as xylene, acetone, and optimal cutting temperature compound (O.C.T.), a protocol was adapted to perform inclusion, using mineral oil instead of xylene during the clearing step (36). After serial dehydration in isopropanol, and then serial treatment with mineral oil diluted in isopropanol, cells were embedded in paraffin. Cuts were made at 5 μm (for analyses at lower magnifications) and at 10 μm (for analyses at higher magnifications). Deparaffinization was performed with progressive baths in alkaline detergent (pH = 10) at 90° and then in hot (90°) and warm water, successively. Afterward, the slides were incubated for 1 min in hematoxylin, rinsed for 5 min in warm water, and another 5 min in running water. Then stained with eosin for 10 min, after which they were washed in warm water for 1 min, dried and mounted in Entelan.

### 2.13 Cultured meat cooking process

The stacked chicken cell construct was subjected to cooking. Following a 6-day culture period, the excess culture medium was removed from the microtissue, which was then carefully transferred, wrapped in film, and stored in a refrigerator overnight. To prepare the cultured meat for consumption, the tissue was placed in a Teflon pan positioned on a hotplate with a pre-heated surface at the temperature of 200°C, and it was cooked in olive oil until it achieved a golden brown appearance. The cooking process was repeated with two independent stacking preparations.

### 2.14 Statistical analysis

Statistical analysis was performed using GraphPad Prism version 8.0.0 for Windows, GraphPad Software, San Diego, California USA, www.graphpad.com. All assays were conducted using three technical replicates (triplicates) and at least 2 independent experiments (biological replicates) were performed. All quantitative data are presented as means+_ standard deviations. Before statistical comparisons, normality (Gaussian distribution) of the data was tested through Prism collumn analysis using the Shapiro–Wilk normality test. Comparative analysis between two groups evaluating only one categorical variable was performed using a parametric Student’s t-test. Analysis of three or more groups with two independent variables was performed using a two-way analysis of variance ANOVA with Bonferroni’s multiple comparison test against indicated control(s). The respective statistical tests are specified in the figure captions. Differences were deemed significant at *p* < 0.05.

## 3 Results

### 3.1 Characterization of random and aligned CAN

**Figure 1:**
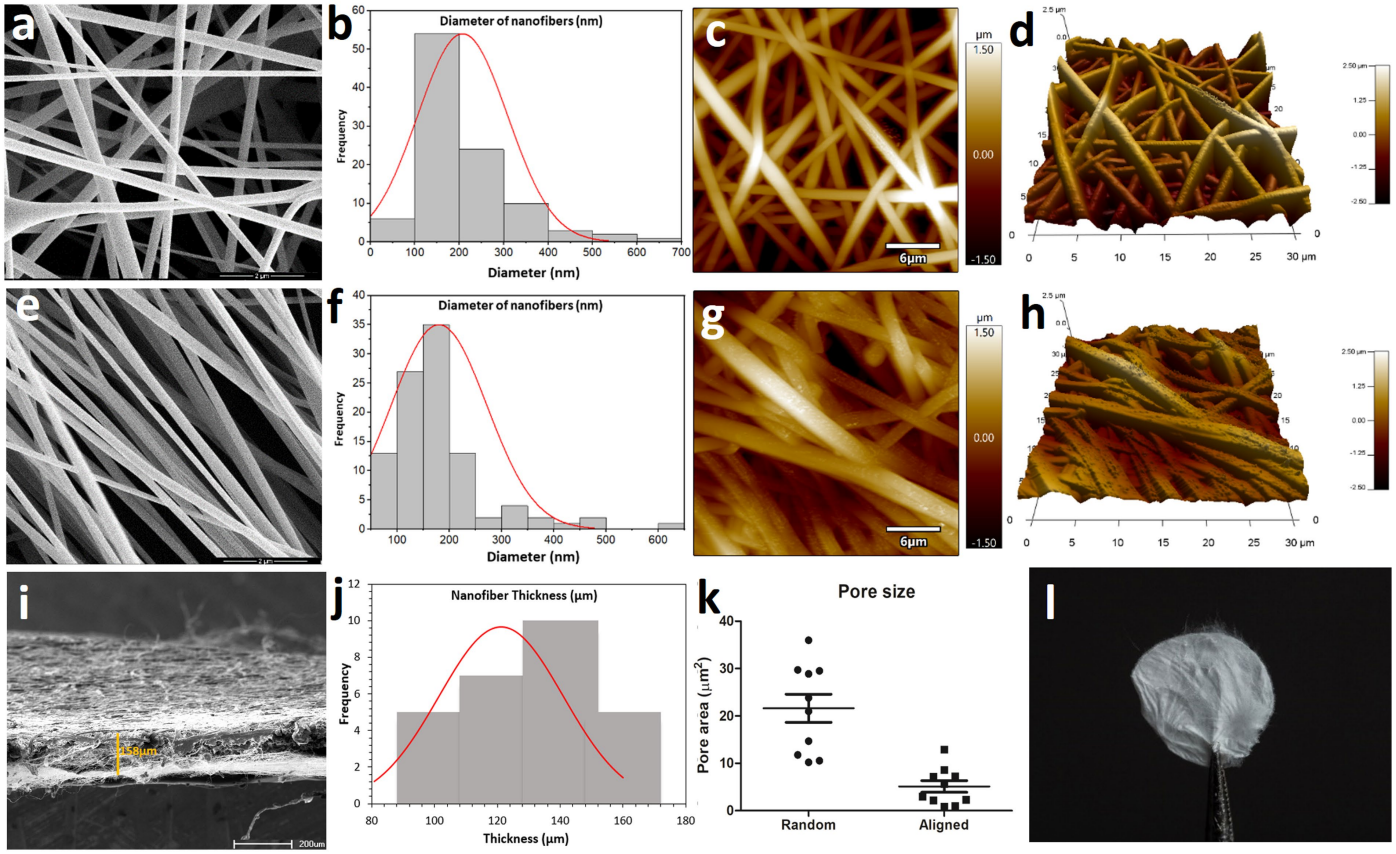
Topographical and surface analysis of random CAN (rCAN) and aligned CAN (aCAN). Morphological evaluation of rCAN and aCAN through scanning electron microscopy (SEM). **(A)** Random and **(E)** aligned CANs SEM images at 50,000x magnification. Scale, 2 μm; Diameter distribution of **(B)** random and **(F)** aligned CAN (*n* = 200 independent fibers examined over three independent samples). Atomic force microscopy (AFM) images of **(C)** random and **(G)** aligned CANs. Scale, 6 μm; 3D AFM images of **(D)** random and **(H)** aligned CAN (30 μm × 30 μm). **(I)** Thickness of nanofiber mats measured from oblique visualization of 4 independent samples. **(J)** Means and distribution (*n* = 10 independent thicknesses examined over four independent samples) of thickness of CANs. **(K)** Average pore area (*n* = 10 independent pores examined over three independent samples) of rCAN and aCAN. **(L)** Image of the rCAN.

Electrospun fibers offer an alternative to improve ECM mimicry. There are two general arrangements for using electrospun mats - random and aligned nanofibers. Random meshes can be used for applications that require load-bearing capacity in all directions. Mats composed of aligned fibers can be used to provide topographical guidance for cell morphogenesis in anatomical anisotropic tissues (37, 38). Here, we produced random and aligned cellulose acetate nanofibers through electrospinning. Electrospun nanofibers were analyzed in a scanning electron microscope (SEM) and in an atomic force microscope (AFM) (Figure 1). SEM and AFM micrographs showed that random CAN (rCAN) presents a highly porous structure with uniform and smooth morphology, ideal for cell infiltration and scaffold colonization (Figures 1A,C,D). Aligned cellulose acetate nanofibers (aCAN), as expected, appeared as a highly aligned mat, but consequently were poorly porous (Figures 1E,G,H). Fiber diameter analysis showed that random and aligned nanofibers had a similar profile, with the majority of the fibers between 100 and 200 nanometers (Figures 1B,F). In addition, the analysis of substrate porosity confirmed that rCAN presented more and larger pores, with a mean area diameter of 21 μm^2^, whereas aCAN presented a mean area diameter of only 4 μm^2^ (Figure 1K). Lastly, oblique SEM images allowed the analysis of the scaffold thickness, with a larger distribution around 120 and 140 micrometers (Figures 1I,J). Apparent features of the nanofiber at a macro level are shown in Figure 1L.

### 3.2 CAN are biocompatible with different myoblast cell lines and promote adhesion without the need for any previous coating

**Figure 2:**
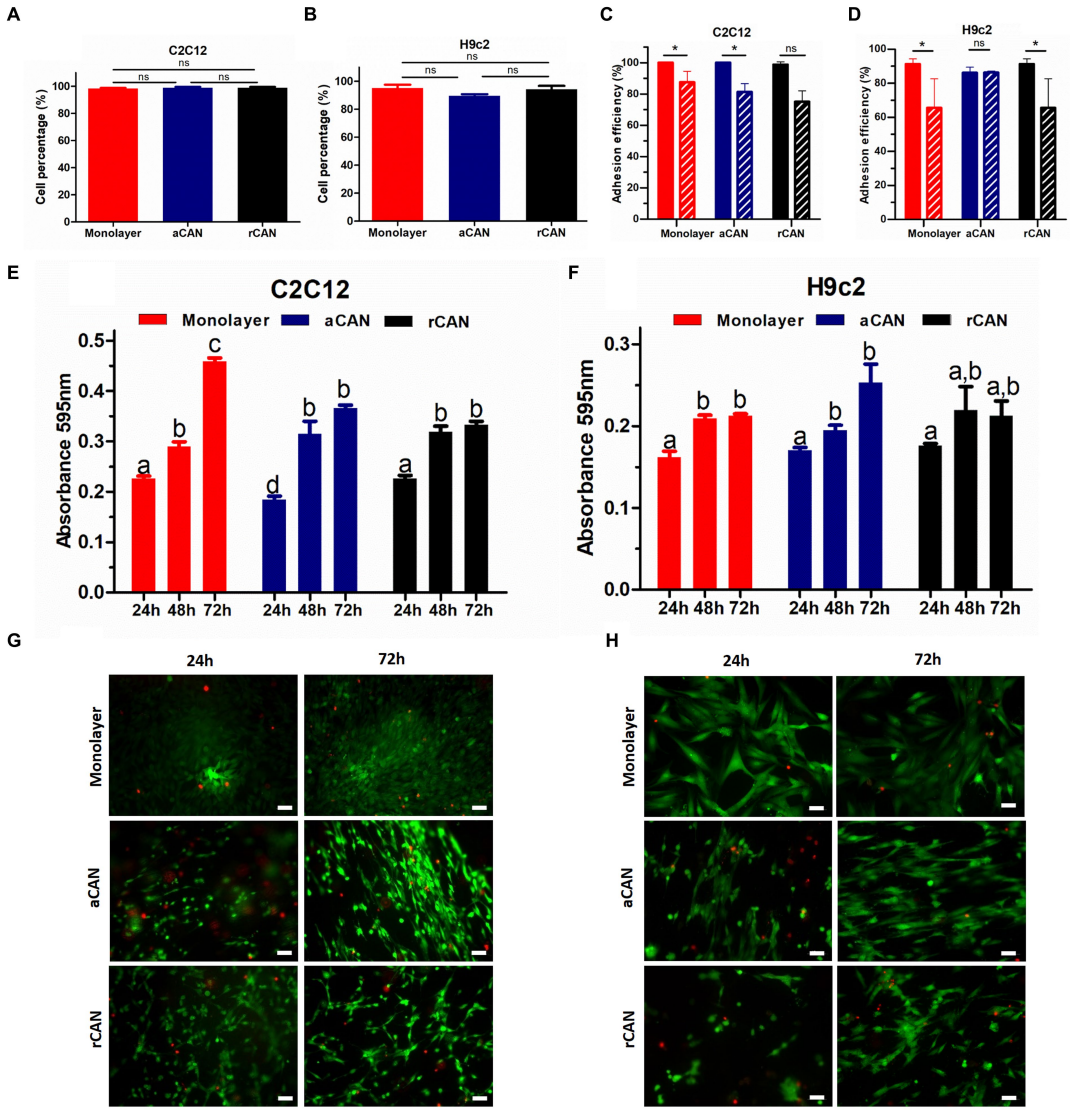
C2C12 and H9c2 myoblasts adhesion, viability and proliferation in rCAN and aCAN. Evaluation of cell-biomaterial interaction. Seeding efficiency (%) of C2C12 **(A)** and H9c2 **(B)** myoblasts, 24 h after plating. **(C)** C2C12 and **(D)** H9c2 adhesion efficiency (%) onto the monolayer (control), rCAN and aCAN in the absence of fetal bovine serum (FBS). MTT assay of **(E)** C2C12 and **(F)** H9c2 myoblasts plated onto the monolayer (control), aCAN or rCAN along 24, 48, and 72 h of culture. Live/Dead staining of **(G)** C2C12 and **(H)** H9c2 myoblasts on the monolayer, aCAN and rCAN at 24 and 72 h. Live cells are shown in green (calcein-AM) and dead cells in red (ethidium homodimer-1). Scale bars: 50 μm. Data presented as mean ± SEM. Asterisks and different letters above bars indicate statistically significant differences, *p* < 0.05 [**(A–D)** Student’s *t*-test; **(E,F)** Two way ANOVA].

**Figure 3:**
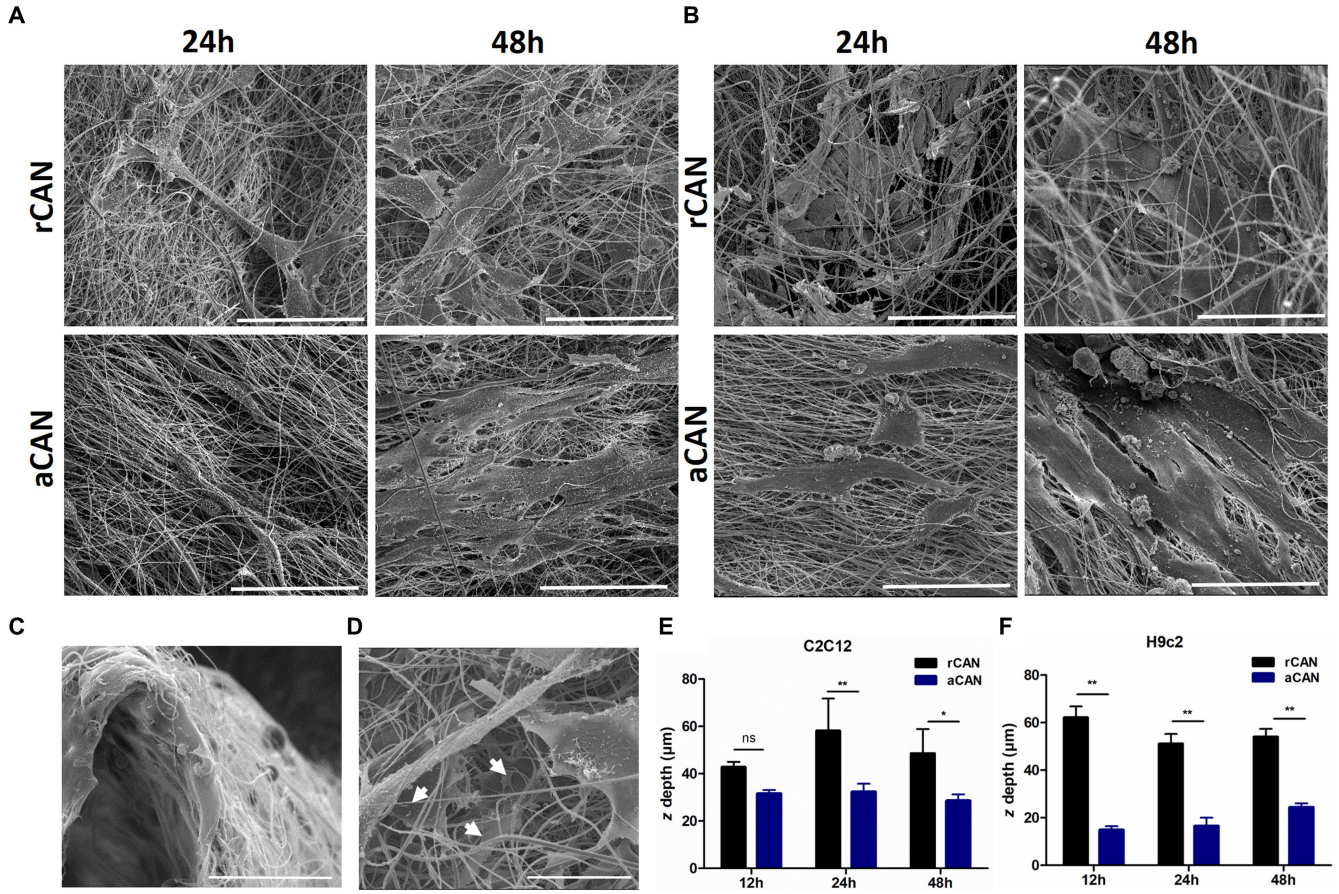
Scanning electron microscope (SEM) images and cell infiltration of C2C12 and H9c2 cells growth on aCAN and rCAN. SEM images of **(A)** C2C12 and **(B)** H9c2 myoblasts cultivated onto aCAN and rCAN after 24 and 48 h of culture at 1,200x magnification. Scale, 50 μm; **(C–F)** Cell infiltration into the scaffolds. **(C)** Cross-view SEM image of H9c2 myoblasts plated on rCAN. Scale, 50 μm; **(D)** SEM images showing infiltration of H9c2 cells in rCAN (white arrows indicate adhered cells along the rCAN *z*-axis). Scale, 50 μm; Cell infiltration quantification on aCAN and rCAN, through analysis of the range of actin fluorescence of **(E)** C2C12 and **(F)** H9c2 myoblasts in the z-axis using confocal microscopy. Results are presented as mean ± SEM. Asterisks indicate statistically significant differences, *p < 0.05 e ** *p* < 0.01 (Two-way ANOVA).

Although it had been shown before that C2C12 could be cultivated into rCAN (17), its efficiency in cell adhesion, proliferation, and colonization was not previously evaluated, neither it was compared to aCAN. To better characterize the suitability of r and aCAN for future cultivated meat purposes, we evaluated the attachment and growth of different lineages of muscle cells into these substrates. C2C12 and H9c2 were seeded onto the scaffolds or onto a glass coverslip, as control (Figure 2). To evaluate cell-biomaterial interaction and the success of the cell’s adhesion to the substrate, seeding efficiency was calculated. For this, supernatants of C2C12 and H9c2 culture were recovered 24 h after cell plating (Figures 2A,B). Similar results, close to 100% efficiency, were seen in all groups, indicating comparable adhesion rates between rCAN or aCAN and the two-dimensional (glass coverslip/monolayer) culture. Additionally, to assess the contribution of fetal bovine serum (FBS) in cell adhesion, as these cultures were made in the presence of 10% FBS, the same analysis was performed, but in the absence of serum (Figures 2C,D). A slight reduction in adhesion was detected in all substrates. In the absence of serum, C2C12 adhesion efficiency in the monolayer and rCAN was 75%, while in aCAN, it was 81%. For H9c2, there was a more significant reduction in cell adhesion without FBS for the monolayer and rCAN (~65% adhesion rate) when compared to aCAN (~82% adhesion rate). To assess overall cellular aspect upon serum deprivation, we performed actin cytoskeleton staining. C2C12 and H9c2 cells cultured without serum had a better morphology when cultured in CAN than on the monolayer (Supplementary Figure 1). These results show that the cells presented good adhesion on CAN, even in the absence of FBS. Morphological analysis through SEM indicated that, just 6 h after seeding, myoblasts could already reach a good adhesion in CAN and that their morphology was completely different from the cells plated onto the coverslip (monolayer) (Supplementary Figure 2). Analysis in later time points, upon 24 and 48 h of culture, showed a more elongated phenotype for H9c2 and C2C12 cells plated onto rCAN and aCAN, when compared to monolayer cultures (Figures 3A,B). Also, cells seemed to infiltrate deeper into the rCAN as compared to the aligned ones (Figures 3C,D). To better address that, we performed a *z* axis analysis on a confocal microscope, upon 12, 24 and 48 h of culture. In general, both cells did present a much better infiltration in the random scaffolds at all times analyzed, compared to the aligned one (except for C2C12-12 h). At 24 h of culture, C2C12 cells reached a depth of 58 μm and 32 μm for rCAN and aCAN, respectively (Figure 3E), while H9c2 cells’ infiltration was 56 μm and 19 μm for rCAN and aCAN, respectively (Figure 3F). Viability and proliferation profiles of the cells in the different substrates were also accessed through MTT assay along culture time. Results obtained after 24 h of culture showed that cell viability in the different scaffolds (rCAN or aCAN) was similar to those of the monolayer for both cell lines (Figures 2E,F). As expected for C2C12 monolayers, upon culture time (48 and 72 h) an increase in MTT values was observed, indicating cell proliferation. On the other hand, in both rCAN and aCAN, MTT values increased only up to 48 h of culture, remaining steady after that. Since no relevant cell death between 24 and 72 h was detected through live/dead assay (LIVE/DEAD™ Viability/Cytotoxicity Kit - Invitrogen) (Figure 2G), this stagnation indicated that cell proliferation was halted. For H9c2 cells, MTT assays showed that cell growth was similar in the monolayer and in the aCAN. On these substrates, the cells maintained the proliferation rate until the second day, after which proliferation ceased. On the other hand, no cell growth was observed for H9c2 plated on rCAN, since the first day of culture. Again, no relevant cell death was detected using the live/dead assay (Figure 2H). Since cell cycle arrest usually occurs upon cell differentiation, the results obtained were suggestive of myoblast maturation, even in the absence of a differentiation media. *In vitro*, myoblasts decrease mitosis and undergo incipient myogenesis when reaching cell confluency. To understand whether confluency could account for the observed early proliferation arrest, two different C2C12 and H9c2 cell densities (1×10^5^ and 5×10^5^) were seeded onto CAN. A similar and low proliferation profile was observed for both cell densities in CAN, whilst a completely different, high proliferation profile, was obtained for the monolayer culture (Supplementary Figure 3), confirming that the arrest in cell proliferation was not due to confluence and that this behavior could be due to scaffold’s topography.

### 3.3 CAN alone triggers myoblast differentiation without the need for any additional factors

**Figure 4:**
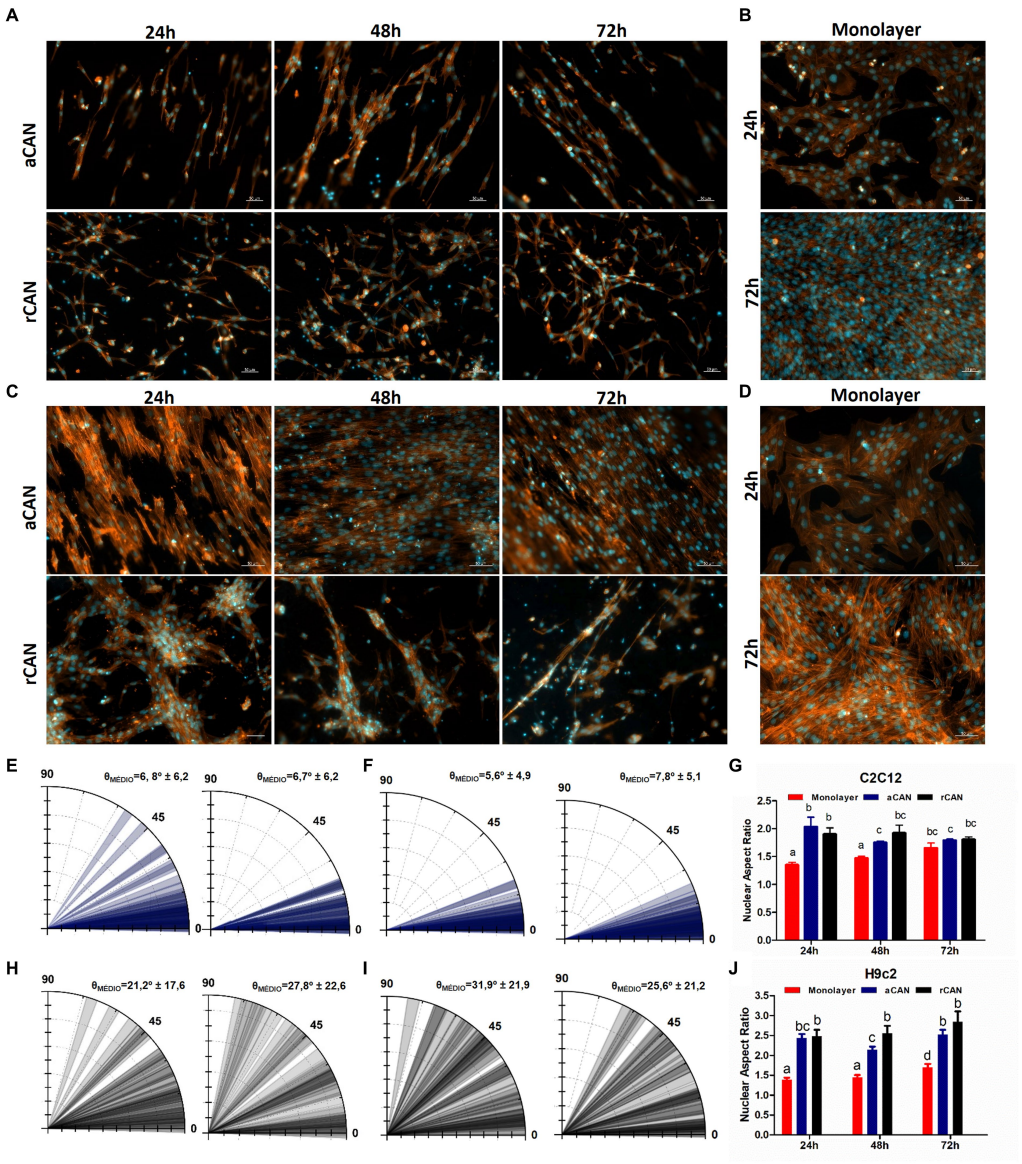
Evaluation of differentiation phenotype by morphological analysis. C2C12 and H9c2 morphology upon culture onto the monolayer, aCAN, and rCAN. Actin fluorescence microscopy images of C2C12 **(A,B)** and H9c2 **(C,D)** myoblasts plated onto aCAN or rCAN along 3 days of culture and monolayer **(B,D)** at 24 and 72 h. Cell nuclei are labeled with DAPI (blue) and the actin filaments are labeled with Phalloidin–Alexa Fluor 546 (red). The scale bar corresponds to 50 μm. 10X magnification. Polar graphs of C2C12 **(E,H)** and H9c2 **(F,I)** at 24 h (left) and 72 h (right). Blue bars represent aCAN and black bars represent rCAN. θ displays the mean angle orientation of cells coupled to the experiment standard deviation (50 reads for two independent experiments). Nuclear cell aspect ratio at 1, 2, and 3 days of culture for C2C12 **(G)** and H9c2 **(J)** cultured on monolayer (red bars), aCAN (blue bars) and rCAN (black bars). Results are presented as mean ± SEM. Different letters above bars indicate statistically significant differences, *p* < 0.05 (Two-way ANOVA).

In stem cell biology, either a cell is proliferating or it is differentiating (39). Because we spotted a brake in proliferation, we wondered whether the cells were in fact undergoing myogenesis in the scaffolds without any differentiation media. During this process, myoblasts become longer and thinner cells, after which they align and start to fuse to form multinucleated myotubes. To verify whether this was happening with cells cultured onto CAN, we first checked cell morphology through actin staining, comparing cells cultured as monolayers or onto the scaffolds, for 3 days in growth medium (GM) (Figure 4). In the first day of monolayer culture (Figures 4B,D) it was possible to observe that C2C12 and H9c2 presented a flat morphology, displaying one nucleus and being homogeneously distributed throughout the surface of the coverslip. After 72 h, H9c2 started showing a more elongated and aligned morphology. This was even more evident for C2C12 cultures, a phenotype suggestive of skeletal muscle differentiation (Figures 4B,D). Likely, these resulted from the high cell density of the monolayers at this time point, since myoblast cell–cell contact triggers differentiation (40). For the cells cultured onto the CAN, due to their three-dimensional architecture, a distribution along the different planes of the substrate could be observed (Supplementary Videos 1–4). In these 3D substrates (Figures 4A,C), cells displayed a longer, tube-like morphology. Already in the first 24 h, small clusters with an ordered arrangement could be seen. After 48 h, cells plated onto both scaffolds became thinner and more elongated, showing more space between cell clusters. This behavior was even more pronounced at 72 h, with many myotube-like structures evidenced in both scaffolds for both cell lines. Cell alignment was more evident in aCAN, as displayed by the actin labeling (Figures 4A,C), and confirmed using the angle tool, as shown in the polar graphs (Figures 4E,F). On rCAN, cells do align to form myotubes, but in heterogeneous directions (Figures 4H,I). To better characterize cells’ morphological phenotype, we performed morphometric analysis by measuring nuclear aspect ratio (NAR) (nuclear length over nuclear width), once the nuclear shape becomes more oval during myogenesis, following cell elongation (Figures 4G,J). As expected, NAR for both C2C12 and H9c2 was smaller for cells in monolayers, as compared to both CAN on the first 2 days of culture. At 72 h, likely because of high cell density in the monolayer, C2C12 showed no difference in NAR for all groups (monolayer, rCAN and aCAN). For H9c2, on the other hand, the difference in NAR between cells plated onto CAN and monolayer remained at 72 h. On the scaffolds, H9c2 presented a more elongated nuclei morphology than those on the monolayer, suggestive of cell differentiation. These results reinforced the role of CAN in the induction of differentiation, as suggested by the cell’s proliferative data.

**Figure 5:**
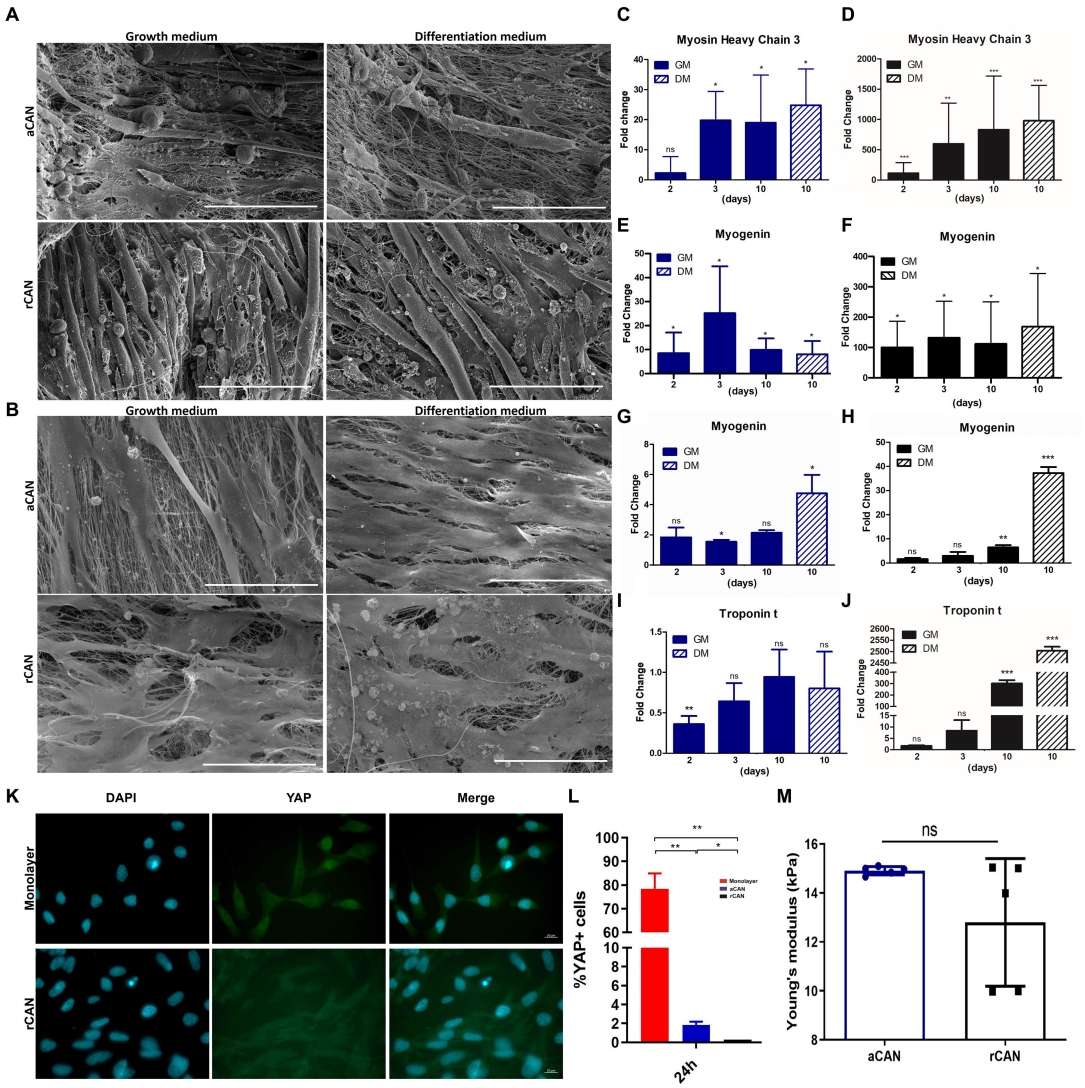
Morphological and molecular differentiation phenotype with growth medium (GM) and differentiation medium (DM). SEM images of **(A)** C2C12 and **(B)** H9c2 myoblasts cultivated onto aCAN and rCAN in GM (10 days) and DM (3 days in GM + 7 days in DM) at 300x magnification. Scale, 200 μm; Relative expression levels of differentiation genes in C2C12 and H9c2 cells cultured onto monolayer (control), aCAN, and rCAN. Relative expression levels of **(C,D)** *Myosin Heavy Chain 3* and **(E,F)** *Myogenin* in C2C12 myoblasts plated onto **(D,F)** rCAN and **(C,E)** aCAN. Expression gene levels from 2, 3, and 10 days in GM or 3 days in GM plus 7 days in DM were compared to expression levels at 1 day in GM. Relative expression levels of **(G,H)** *Myogenin* and **(I,J)** *Troponin t* in H9c2 myoblasts plated onto **(H,J)** rCAN and **(G,I)** aCAN. Expression gene levels from 2, 3, and 10 days in GM or 3 days in GM plus 7 days in DM were compared to expression levels at 1 day in GM. Significative data was obtained by REST2009 software, using *p* < 0.05. **(K)** Fluorescence microscopy of the local distribution of the YAP protein in C2C12 cells spreading on monolayer and rCAN. Cells were stained with anti-YAP (green) and nuclei were stained with DAPI (blue), scale bar corresponds to 50 μm. **(L)** Quantification of the ratio of positive YAP nucleus cells of C2C12 myoblasts cultured for 24 h in GM, based on stacks of fluorescence images like the ones shown in letter k. **(M)** Young’s modulus (E) of the random and aligned CANs (mean ± SD). Results are presented as mean ± SEM. Asterisks indicate statistically significant differences, *p < 0.05 e **p < 0.01 (**C–L** Two-way ANOVA; **M** Student’s *t* test).

To better address this hypothesis, we performed experiments with cells cultured either in growth (GM) or in differentiation medium (DM). SEM images showed that both myoblasts, grown under DM on both CAN, were very similar to those cultured under GM (Figures 5A,B). Additionally, the mRNA levels of muscle differentiation markers were evaluated. For C2C12, *Myosin Heavy Chain 3* (*MyH3*), a mysosin expressed during muscle development, was used as a differentiation marker, since its levels have shown to be enhanced upon induction of differentiation in culture (41). For H9c2, we quantified the expression of *cardiac troponin T* (*cTnT*), a structural protein found in the contractile apparatus of cardiac myocytes. This protein is known to be expressed upon treatment with 10 nM all-trans-retinoic acid (all-trans-RA) in culture, as a consequence of their differentiation into the cardiac phenotype (32). *Myogenin* (*MyoG*) was used for both C2C12 and H9c2 since it is expressed in both ventricular myocardium and skeletal muscle during the beginning of cell differentiation (42). For C2C12 plated onto aCAN and then cultured in the presence of DM (positive control), a 20-fold increase in *MyH3* expression level, in relation to day one of culture, was detected, indicating cell differentiation (Figure 5C). The same increase in *MyH3* expression was observed at day 3 and 10 of C2C12 culture in GM (Figure 5C). An increase in *MyH3* expression level was also observed for C2C12 plated onto rCAN and kept in GM (Figure 5D). However, for cells plated onto rCAN, the fold change was not only higher when compared to aCAN, but also occurred as early as day two of culture (around 100 times at day two of culture and 800 times higher at day three and ten, when compared to day one of culture). Also, *MyH3* expression level at day 10 of culture in growth or differentiation media were similar. Expression of *MyoG* was also increased in C2C12 plated onto aCAN or rCAN and kept in GM, since day two of culture in relation to day one (Figures 5E,F). However, as observed for the expression level of *MyH3*, the fold change was higher for cells cultured onto rCAN (~100 at day 2 and 600-fold on the following days) in relation to aCAN (~10-fold for all days of culture). For H9c2 plated onto aCAN and kept in GM, troponin T was found to be downregulated (~0.4-fold) at day two of culture, in relation to day one (Figure 5I). No increase in the levels of Troponin t was observed for all other time points or for cells cultured in DM. An increase of ~1.8-fold in *MyoG* expression could be observed only at day three in GM, compared to day one, while an increase of ~5-fold was observed when these cells were plated onto aCAN and then cultivated in DM (Figure 5G). For H9c2 plated onto rCAN and kept in GM, as for C2C12, we found a more significant upregulation of differentiation genes (Figures 5H,J). An increase in troponin T mRNA levels was observed at day 10 of culture, by >300 and 2,500-fold, in GM and DM, respectively. For *MyoG,* an increase of 8 and 40-fold was observed for cells maintained in GM and DM, respectively. Altogether, these results support the idea of cell differentiation in rCAN, without any additional external stimuli, and highlight the special ability of rCAN in triggering myogenesis on both H9c2 and C2C12 cells, regardless of DM administration.

### 3.4 CAN induced-myogenesis involves YAP/TAZ mechanotransduction signaling activated through substrate stiffness

A major goal of MTE is to generate muscle that has features similar of native tissue. Our previous results explored the ability of aCAN and rCAN in triggering the differentiation of myoblast into myotubes in a and rCAN. Despite the good results found for both nanofibers, rCAN showed a more pronounced myogenesis process. Since myoblast proliferation and differentiation are sensitive to physical and mechanical cues (43–46), we sought to understand the differences in the stiffness between the substrates. For that, we characterized the mechanical properties of the nanofibers using atomic force microscopy (AFM). We found that Young’s modulus (E) has similar values for aCAN and rCAN, which was measured as E = 14.9 + −0.2 kPa for aCAN and as E = 12.8 + −2.6 kPa for rCAN (Figure 5M). It is known that substrates that resemble muscle-like stiffness may induce myogenic gene expression, while rigid substrates (e.g., glass) could delay, if not stop, differentiation (47). As substrate stiffness of ~8–16 kPa was shown to be ideal for the myogenesis of mouse C2C12 cells (47), our nanofibers (aligned and random) showed optimal stiffness for the differentiation of muscle cells.

It is recognized that cells are able to translate mechanical inputs to intracellular biochemical and biophysical signals in a process called mechanotransduction (48). The Hippo signaling pathway comprehends multiple crosstalks and interactions of proteins upstream of its final player, the Yes-associated protein (YAP), whose localization can be modulated by mechanical cues that are sensed through cytoskeleton modifications (49–51). When activated, YAP is located inside the nucleus, targeting genes related to proliferation and survival. When inactivated, it is phosphorylated and translocated to the cytoplasm, abrogating its transcriptional effects (52). In muscle biology, studies have pointed that YAP/TAZ is involved in myogenesis and increase in YAP phosphorylation is important for cell differentiation (53, 54). Thus, we investigated whether the differentiation triggered by both aCAN and rCAN was coordinated by YAP and the Hippo pathway. Through immunofluorescence, we showed that YAP was preferentially located inside the nuclei of cells plated onto the coverslip, whereas very few YAP positive nuclei staining was observed for cells cultured in aCAN and no YAP-positive nuclei was detected on cells cultured in rCAN (Figures 5K,L), suggesting that CAN, especially rCAN, was able to prompt mechanotransduction processes coordinated by the Hippo pathway. Our data indicate that cultures on more elastic substrates favor muscle cell differentiation through YAP/TAZ signaling.

### 3.5 rCAN may be stacked to produce thick tissue samples

**Figure 6:**
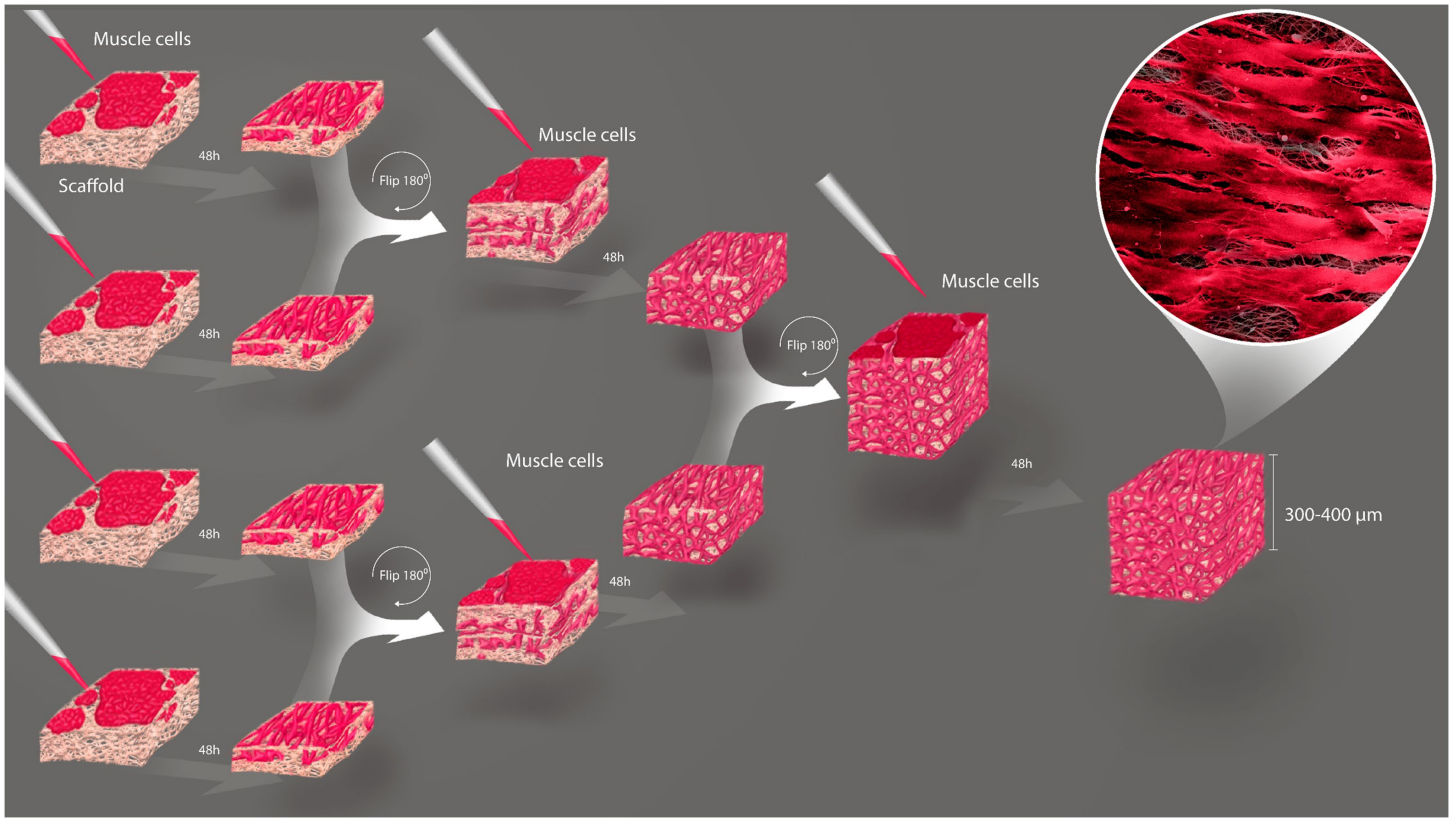
Establishment of the stacking methodology. Schematic illustration of three-dimensional tissue formation by stacking cells grown on rCAN. 5 × 10^5^ cells are grown 2 days in GM and then stacked with surfaces facing each other. More 5 × 10^5^ cells are plated on the new-formed surface and those 2-layer constructs are incubated another 2 days, when they are stacked to each other with the same prior configuration. 5 × 10^5^cells are again seeded above the construct and let in culture for more 2 days.

**Figure 7:**
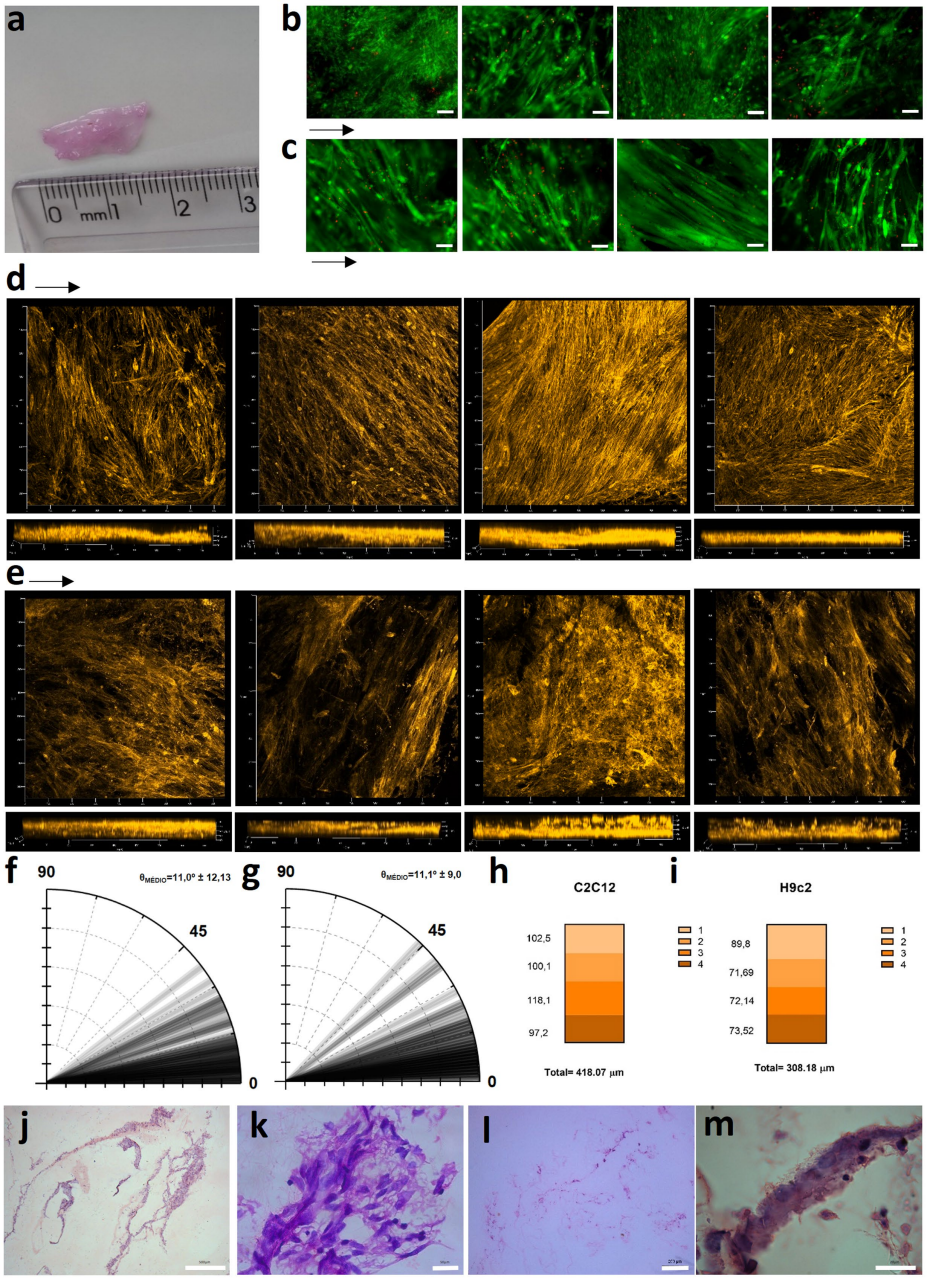
Stacked tissue characterization. **(A)** Size of the 4-layer stacked muscle tissue. Live-dead assay images for **(B)** C2C12 and **(C)** H9c2 onto rCAN after the 4-layer stacking process, from the top (left) to the bottom layer (right). Actin staining images of x, y, and z axis are shown for **(D)** C2C12 and **(E)** H9c2 myoblasts grown for 6 days in GM, from the top (left) to the bottom layer (right). Polar graphs for **(F)** C2C12 and **(G)** H9c2 cultured for 6 days in GM (*n* = 80 cells from two independent constructs). Cell infiltration quantification on each layer of the 4-layer stacking, through analysis of actin fluorescence of **(H)** C2C12 and **(I)** H9c2 myoblasts in the z-axis using confocal microscopy, with a total estimated depth of cells infiltrated in the 4-layers stacked construct. Hematoxylin and eosin staining for **(J, K)** C2C12 and **(L, M)** H9c2 of the engineered tissue.

One hurdle of MTE is the obtention of thick tissues, due to low nutrient and gas diffusion that compromises cell viability inside the construct (27). Aiming to circumvent this, we stacked sheets of porous rCAN cultivated with C2C12 or H9c2, and characterized cells viability and morphology throughout the construct. The assembly method for the nanofiber stacks is shown in Figure 6. Stacking started by plating 5×10^5^ cells per nanofiber and maintaining these in GM for 2 days. Then, single layers (cell sheets) were placed on top of one another with the side where the cells were plated facing each other. Another 5×10^5^ cells were plated on the top of the stacked cell sheets and maintained in GM for another 2 days. This process was repeated with the two layers stacked and left for two more days in GM, totaling 6 days in culture and 4 layers stacked. It is important to point out that different stacking configurations were tested to find the best arrangement of the construct, as shown in Supplementary Figure 4. After this process, the cell sheets were separated, either fixed or processed for viability and morphological evaluation. Live-dead assay on all layers showed that myoblasts were highly viable, even in the middle of the construct (Figures 7B,C), proving that the increase in layers did not cause a decrease in cell viability within the construct. After stacking, as shown in the fluorescence images, cells presented an even more elongated and aligned morphology, when compared to cells in a single nanofiber sheet (Supplementary Figure 4A). Stacked cells showed a much more oriented profile (Figures 7D,E), when compared to cells maintained in single rCAN (Figures 4A,C), with a mean angle Θ ~11° for both H9c2 and C2C12 (Figures 7F,G). A pattern of elongated myotube formation was observed, which also indicated an initial differentiation into muscle fibers. From the *z*-stack profiles, we observed that the infiltration of cells through each rCAN depth was ∼100 μm for C2C12 and ~ 70 μm for H9c2 (Figures 7H,I). Also, the cells were very well distributed along the *z*-axis (Figures 7D,E; Supplementary Videos 5–10). These results showed that both cells could optimally infiltrate throughout the nanofiber and that our stacking methodology allowed the formation of tissues ~300–400 μm thick. Hematoxylin and eosin (H&E) staining confirmed cell adhesion, alignment, and myotube formation on both the surface and inside of the rCAN stacking (Figures 7J–M). The final product is shown in Figure 7A, where it is possible to see a construct measuring 2 cm long and 0.5 cm wide, implying the feasibility of the random CAN stacking-engineered muscle fabrication.

### 3.6 rCAN as a platform to the production of cultivated chicken steak

**Figure 8:**
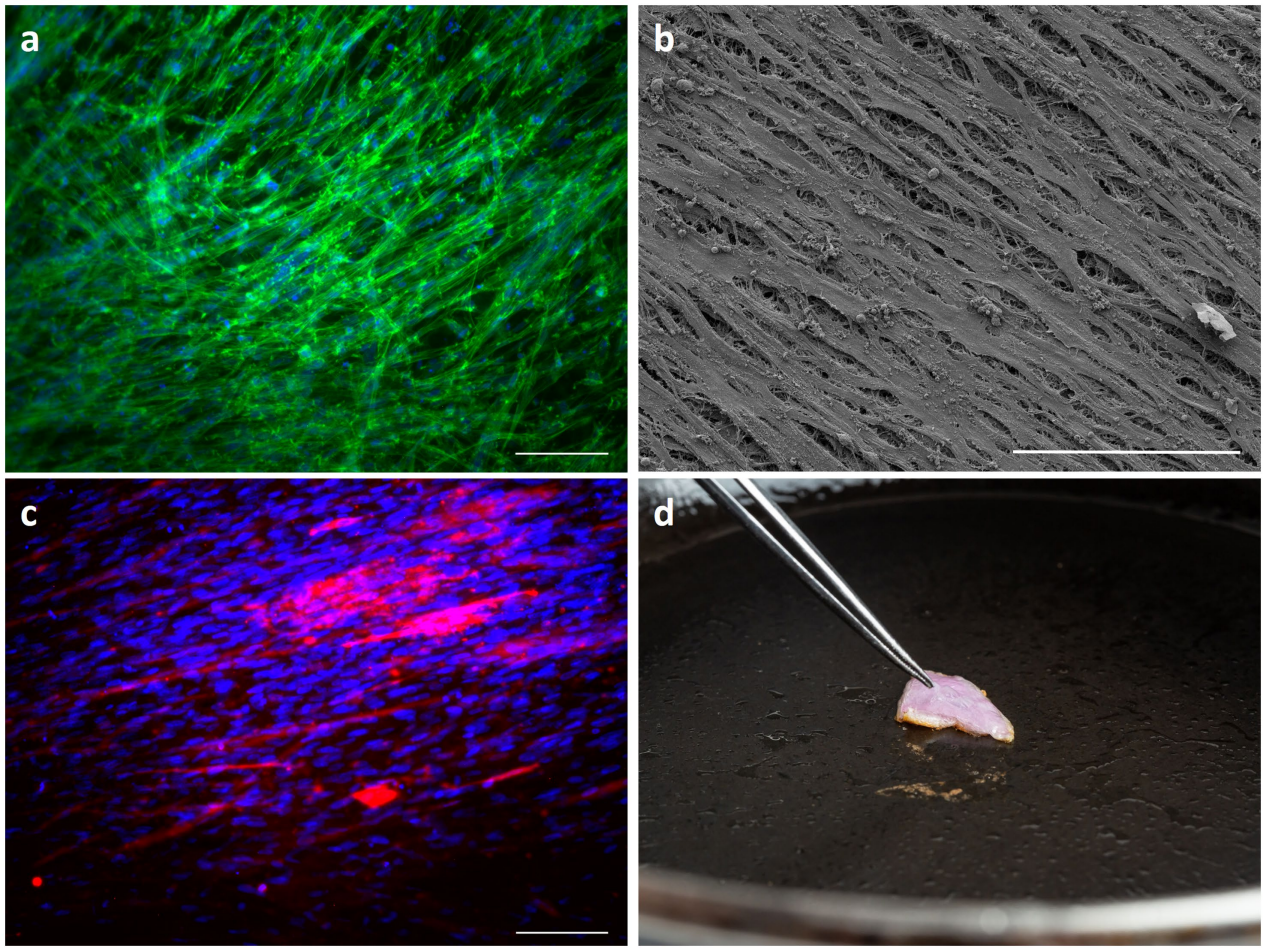
Characterization of engineered chicken skeletal muscle tissue. cSCs morphology through actin staining **(A)** and SEM **(B)** onto rCAN. Cells were cultured, respectively, for 48 h and 72 h in DMEM supplemented with 20% FBS. Cell nuclei are labeled with DAPI (blue) and the actin filaments are labeled with Phalloidin–Alexa Fluor 488 (green). The scale bar corresponds to 50 μm. 10× magnification. For the SEM image, the scale bar corresponds to 100 μm and 1,000× magnification. **(C)** Stacked tissue characterization by Myosin heavy chain immunofluorescence on chicken satellite cells on the fourth layer of the stacked construct. Cells were stained with anti-MF20 (red) and nuclei were stained with DAPI (blue). The scale bar corresponds to 50 μm. 10× magnification. **(D)** Cultivated chicken fried on a griddle pan at 200°C.

To evaluate the capability of the proposed stacking process in generating a whole cut of meat, we cultured chicken satellite cells (cSCs) onto rCAN. cSCs were cultured on single rCAN in growth media. Actin staining and SEM showed high alignment of the cells after 48 and 72 h of culture (Figures 8A,B). The stacking procedure was also performed and aligned chicken muscle fibers were observed as detected by MF-20 immunostaining (Figure 8C), which labels Myosin Heavy Chain. Additionally, the stacked chicken muscle tissue was pan fried (Figure 8D), showing that rCAN is a suitable platform for cultivated meat applications, supporting the cultivation, alignment and differentiation of muscle cells.

## 4 Discussion

In this work we have demonstrated that a scaffold formed by a mesh of fibers randomly distributed (rCAN) is an excellent substrate for MTE and thus for cultivated meat. Applying a stacking-engineered muscle fabrication we were able to produce a thick, tissue-resembling muscle construct, demonstrating that CAN are biocompatible with different myoblast cell lines and promote adhesion without the need for previous coating or surface treatment. Additionally, we demonstrated that the substrate alone was enough to induce cell differentiation using the same medium from the proliferation phase.

As it is well-known, production of cultivated meat faces several technical challenges, many of them related to the complete elimination of all animal components from the manufacturing process. These challenges include the formulation of novel media suitable for cell proliferation and differentiation, as well as the obtention of a 3D structure utilizing biomaterials derived exclusively from plant sources. In this context, the present work contributed to the advancement in sensitive areas of the chain of cultivated meat production. Notably, CAN showed favorable cell adhesion without any functionalization or ECM protein coating. Our results showed that cell adhesion occurred even in the absence of serum which is known to contain cell adhesion factors, such as fibronectin (Figures 2C,D; Supplementary Figure 1) in its composition. Surface chemistry and ECM proteins often influence cell adhesion, with positively charged coatings commonly applied to synthetic scaffolds for improved adhesion (35, 55, 56). The fact that no surface modifications are needed for adhesion to CAN, despite their plant-based origin, simplifies the process and may result in reduced costs during cultivated meat production.

Current literature on MTE generally preconizes scaffolds that already detain a topographical guidance that will allow myoblasts fusion, as well as orientation, thus favoring cell differentiation, to create an aligned tissue that better reproduces the morphology of native muscle tissue (57, 58). Consequently, the substrate of choice has usually an aligned configuration. However, despite the presence of relatively parallel organized collagen I fibers in the muscle ECM, a random-meshed of collagen type III is also present in the muscle endomysium, which confers elasticity to this substrate (21). In this work, we have demonstrated that a scaffold formed by a mesh of fibers randomly distributed (rCAN) is an excellent substrate for MTE and thus for cultivated meat. It not only allowed the formation of aligned bundles of cells, but also was enough to induce cell differentiation, regardless of differentiation media administration (Figures 4, 5). The latter, as we showed, was related to mechanotransduction events, involving Hippo pathway-YAP/TAZ signaling. As mentioned in the results section, YAP’s localization inside the cell can be modulated by mechanical cues that are sensed through cytoskeleton modifications (49–51). Stiffer substrates trigger YAP nuclear localization and thus, cell proliferation, while softer ones prompt YAP inactivation (51). Additionally, it has been shown in the literature that substrate rigidity influences myoblast differentiation (47). The ideal stiffness for adequate myotube formation in C2C12 and H9c2 myoblasts corresponded to that of native skeletal muscle, at approximately 12 kPa (47). Both nanofibers showed stiffness averaging between 12 and 14 kPa, suggesting that the differentiation seen in CAN was led by the similarity of stiffness with native skeletal muscle tissue. These data strongly suggest that our scaffold mechanically triggers YAP signaling that prompts myogenesis, only by its material and architecture, regardless of any additional soluble differentiation factors. This is very desirable for any design of MTE, especially when considering cultivated meat bioprocess, where mitigation of costs is tirelessly sought. In this case, the same medium used during the bioreactor proliferation phase can be employed in the scaffold perfusion differentiation phase, thus avoiding the need for additional expense. This is possible due to the scaffold’s inherent ability to induce differentiation, avoiding the need to develop a distinct culture medium. Nevertheless, it is important to note that rCAN showed a much more pronounced differentiation (Figures 5D,F,H,J). These results might be explained by the porous nature of rCAN mat, in which cells must stretch to anchor in two distant attachment points, as seen in Supplementary Figure 2. During embryonic and postnatal development, tension forces created by tendon insertion are major proponents of myofibril formation (59). In this way, the stretching seen in cells plated onto rCAN might be creating tension forces that resemble native tissue development and better trigger mechanical assets that prompt myogenesis. Besides that, the porous nature of rCAN presents other characteristics desired for MTE scaffolds, such as porosity, mechanical assets, organoleptic properties, and the ability to conduct angiogenesis.

Finally, another important challenge in cultivated meat production is the creation of a 3D structured meat product such as a filet. To achieve this, an appropriate scaffolding approach is required to facilitate cell proliferation, differentiation into the essential cell types, as well as spatial arrangement is needed without compromising cell viability. Here we demonstrated that rCAN, due to its porous nature, not only allowed for deep colonization of the substrate by the muscle cells (Figures 3E,F), but certainly contributed to high cell viability upon layer-by-layer stacking of muscle cell sheets (Figure 7; Supplementary Figure 4). Additionally, cells presented high alignment and an even more elongated morphology, even on the random-meshed CAN (Figure 7; Supplementary Figure 4). This highlights CAN potential as a good scaffold for building a structured thick muscle tissue. Corroborating this hypothesis, we successfully fabricated a 3D chicken muscle tissue, where cells not only remained viable across the entire depth of the tissue following the stacking process, but also presented an even more elongated morphology. We also showed the chicken construct could withstand the cooking process, which is an essential characteristic for cultivated meat. With that, we expect that our model will prime the engineering of edible muscle tissue with the culture of other species such as bovine, pork, or fish cells for cultivated meat applications. However, it is important to note that further investigations into the sensory and nutritional properties are required. Moreover, the random mesh might also be cardinal to biomedical constructs, not only because they better mimic ECM, but because they will also allow angiogenesis and complete implantation of the graft on the patient. In a cultivated meat application, it might also be important for the product’s texture.

## Data availability statement

The original contributions presented in the study are included in the article/Supplementary material, further inquiries can be directed to the corresponding author.

## Ethics statement

All animal procedures were conducted following the instructions of the Ethics Commission on Animal Use of the UFMG (ethical approval 209/2022) and the Brazilian laws for the use of animals in scientific experiments. The study was conducted in accordance with the local legislation and institutional requirements.

## Author contributions

AnS: Conceptualization, Formal analysis, Investigation, Methodology, Visualization, Writing – original draft. JG: Conceptualization, Formal analysis, Investigation, Methodology, Visualization, Writing – original draft. JA: Formal analysis, Investigation, Methodology, Visualization, Writing – review & editing. ItA: Formal analysis, Investigation, Methodology, Visualization, Writing – review & editing. AM: Formal analysis, Investigation, Methodology, Writing – review & editing. AC: Investigation, Methodology, Writing – review & editing. IsA: Investigation, Methodology, Writing – review & editing. AP: Investigation, Methodology, Writing – review & editing, Formal analysis. BN: Investigation, Methodology, Writing – review & editing. JS: Investigation, Writing – review & editing. AlS: Writing – review & editing, Conceptualization, Funding acquisition, Methodology, Resources. EJ: Conceptualization, Resources, Writing – review & editing. LO: Conceptualization, Resources, Writing – review & editing, Funding acquisition, Project administration, Supervision.

## Footnotes

^1^https://doi.org/10.3390/macromol3030031
