# Corrigendum: Random cellulose acetate nanofibers: a breakthrough for cultivated meat production

**DOI:** 10.3389/fnut.2024.1374847

**Published:** 2024-03-15

**Authors:** Ana Elisa Antunes dos Santos, Jorge Luís Guadalupe, Juliano Douglas Silva Albergaria, Itallo Augusto Almeida, Amanda Maria Siqueira Moreira, Aline Gonçalves Lio Copola, Isabella Paula de Araújo, Ana Maria de Paula, Bernardo Ruegger Almeida Neves, João Paulo Ferreira Santos, Aline Bruna da Silva, Erika Cristina Jorge, Luciana de Oliveira Andrade

**Affiliations:** ^1^Department of Morphology, Institute of Biological Science, Federal University of Minas Gerais, Belo Horizonte, Brazil; ^2^Laboratory of Biomaterials, Department of Materials Engineering, Federal Center for Technological Education of Minas Gerais (CEFET-MG), Belo Horizonte, Brazil; ^3^Department of Physics, Institute of Exact Sciences, Federal University of Minas Gerais, Belo Horizonte, Minas Gerais, Brazil

**Keywords:** cellulose acetate, nanofiber, scaffold, muscle tissue engineering, cultivated meat

In the published article, there was an error in the author list, and author Isabella Paula de Araújo (affiliation: Laboratory of Biomaterials, Department of Materials Engineering, Federal Center for Technological Education of Minas Gerais (CEFET-MG), Belo Horizonte, Brazil) was erroneously excluded. The corrected author list appears below.

Ana Elisa Antunes dos Santos^1^, Jorge Luís Guadalupe^1^, Juliano Douglas Silva Albergaria^2^, Itallo Augusto Almeida^1^, Amanda Maria Siqueira Moreira^1^, Aline Gonçalves Lio Copola^1^, Isabella Paula de Araújo^2^, Ana Maria de Paula^3^, Bernardo Ruegger Almeida Neves^3^, João Paulo Ferreira Santos^2^, Aline Bruna Da Silva^2^, Erika Cristina Jorge^1^ and Luciana Oliveira Andrade^1^

The authors apologize for this error and state that this does not change the scientific conclusions of the article in any way. The original article has been updated.

