# Supplementary material for: Random cellulose acetate nanofibers: a breakthrough for cultivated meat production
Source: Front Nutr. 2024 Jan 5;10:1297926. doi: 10.3389/fnut.2023.1297926 (PMC10796801; doi:10.3389/fnut.2023.1297926)
Supplement: Supplementary file 11 [file Data_Sheet_1.docx]

Supplementary Material

Random Cellulose Acetate Nanofibers: A Breakthrough for Cultivated Meat Production

Ana Elisa Antunes dos Santos^1†^, Jorge Luís Guadalupe^1†^, Juliano Douglas Silva Albergaria^2^, Itallo Augusto Almeida^1^, Amanda Maria Siqueira Moreira^1^, Aline Gonçalves Lio Copola^2^, Ana Maria de Paula^3^, Bernardo Ruegger Almeida Neves^3^, João Paulo Ferreira Santos^2^, Aline Bruna da Silva^2^, Erika Cristina Jorge^1^, Luciana de Oliveira Andrade^1*^

^1^Laboratory of Cellular and Molecular Biology, Institute of Biological Science, Department of Morphology, Federal University of Minas Gerais, Belo Horizonte, Brazil

^2^Laboratory of Biomaterials, Department of Materials Engineering, Federal Center for Technological Education of Minas Gerais (CEFET-MG), Belo Horizonte, Brazil

^3^Department of Physics, Instituto de Ciências Exatas, Federal University of Minas Gerais, Belo Horizonte, Minas Gerais, 31270-901, Brazil

† These authors contributed equally to this work and share first authorship

*** Correspondence:**Luciana de Oliveira Andrade
lucianaandrade@ufmg.br

# Supplementary Figures and Tables

## Supplementary Figures

##
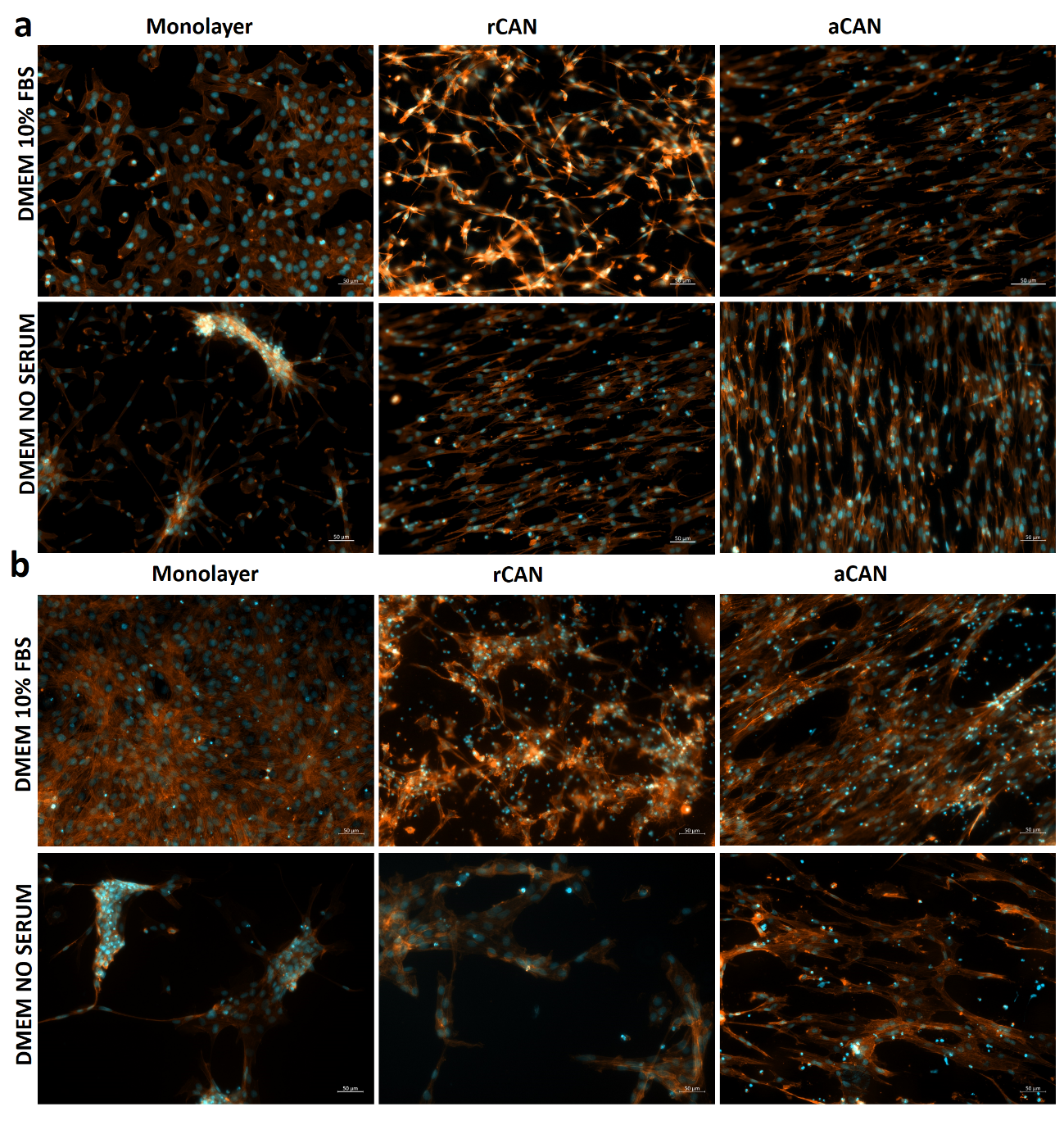


## Supplementary Figure 1. Actin fluorescence microscopy images of (a) C2C12 and (b) H9c2 cells plated on glass coverslips, rCAN and aCAN after 24 hours of culture, in the presence or absence of FBS. Cell nuclei are labeled with DAPI (blue) and the actin filaments are labeled with Phalloidin–Alexa Fluor 546 (orange). The scale bar corresponds to 50 μm. 10X magnification.


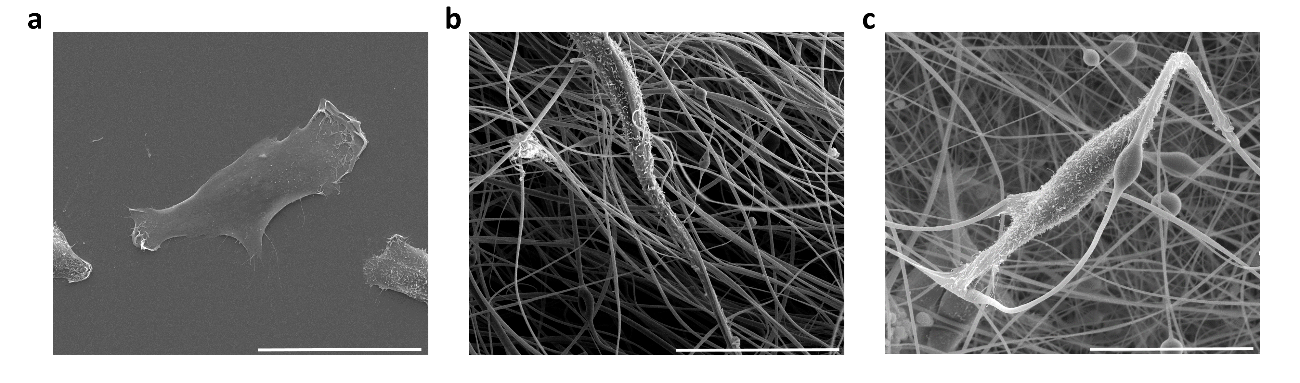


**Supplementary Figure 2.** SEM images of C2C12 cells cultivated onto (a) monolayer, (b) random, and (a) aligned CAN, after 6 hours of plating. Scale bars indicate 50 µm.


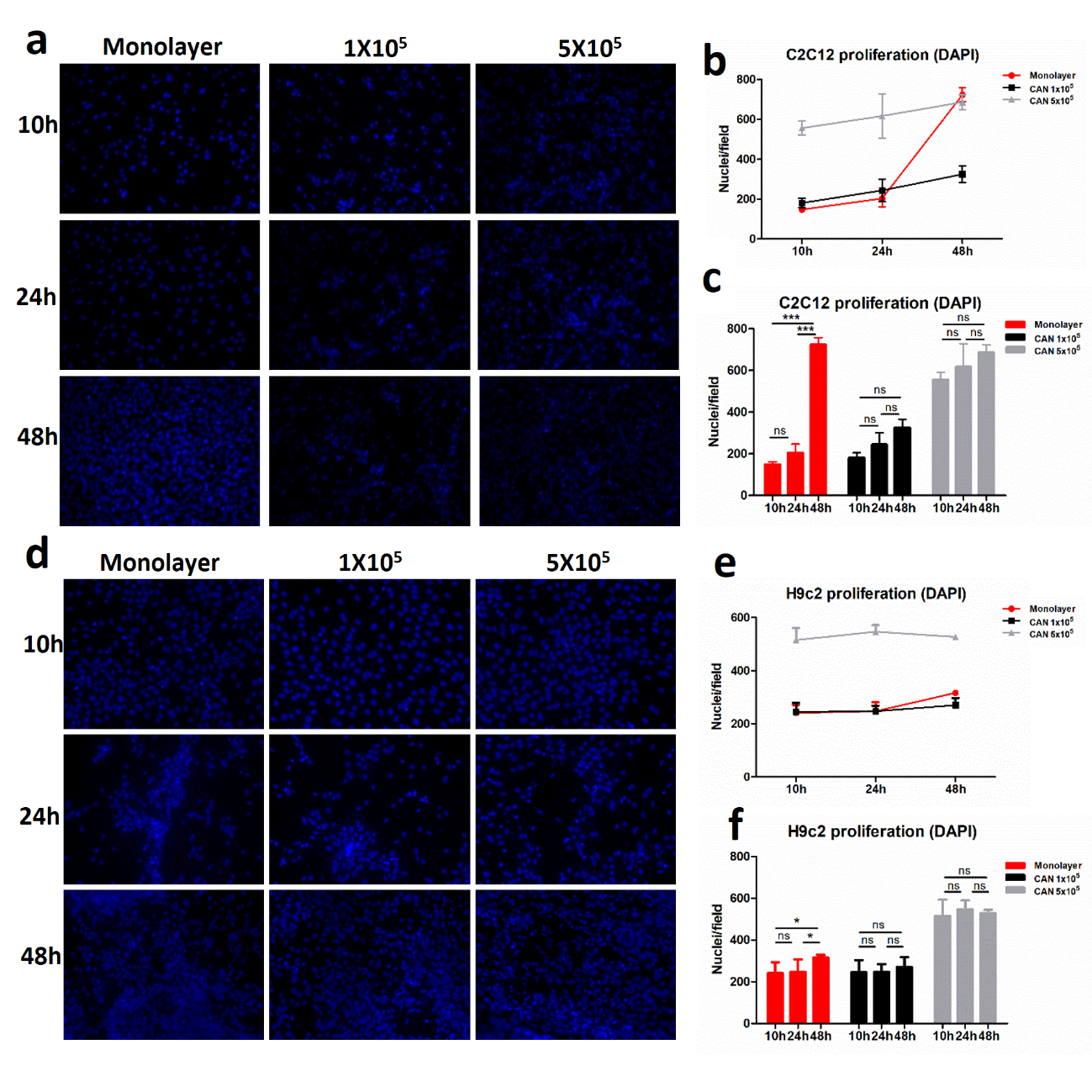


**Supplementary Figure 3.** Nuclei fluorescence microscopy images of (a) C2C12 and (d) H9c2 myoblasts plated on glass coverslips, rCAN, and aCAN after 10, 24, and 48h for C2C12 and H9c2, with different concentrations of cells. 1x10^5^ cells were plated on glass coverslips and 1x10^5^ and 5x10^5^ cells were plated on rCAN and aCAN. The scale bar corresponds to 50 μm. 10X magnification. Nuclei quantification of (b and c) C2C12 and (e and f) H9c2 in curve and bar plots. Asterisks indicated *p<0.05; ***p<0.001 (Two-way ANOVA).


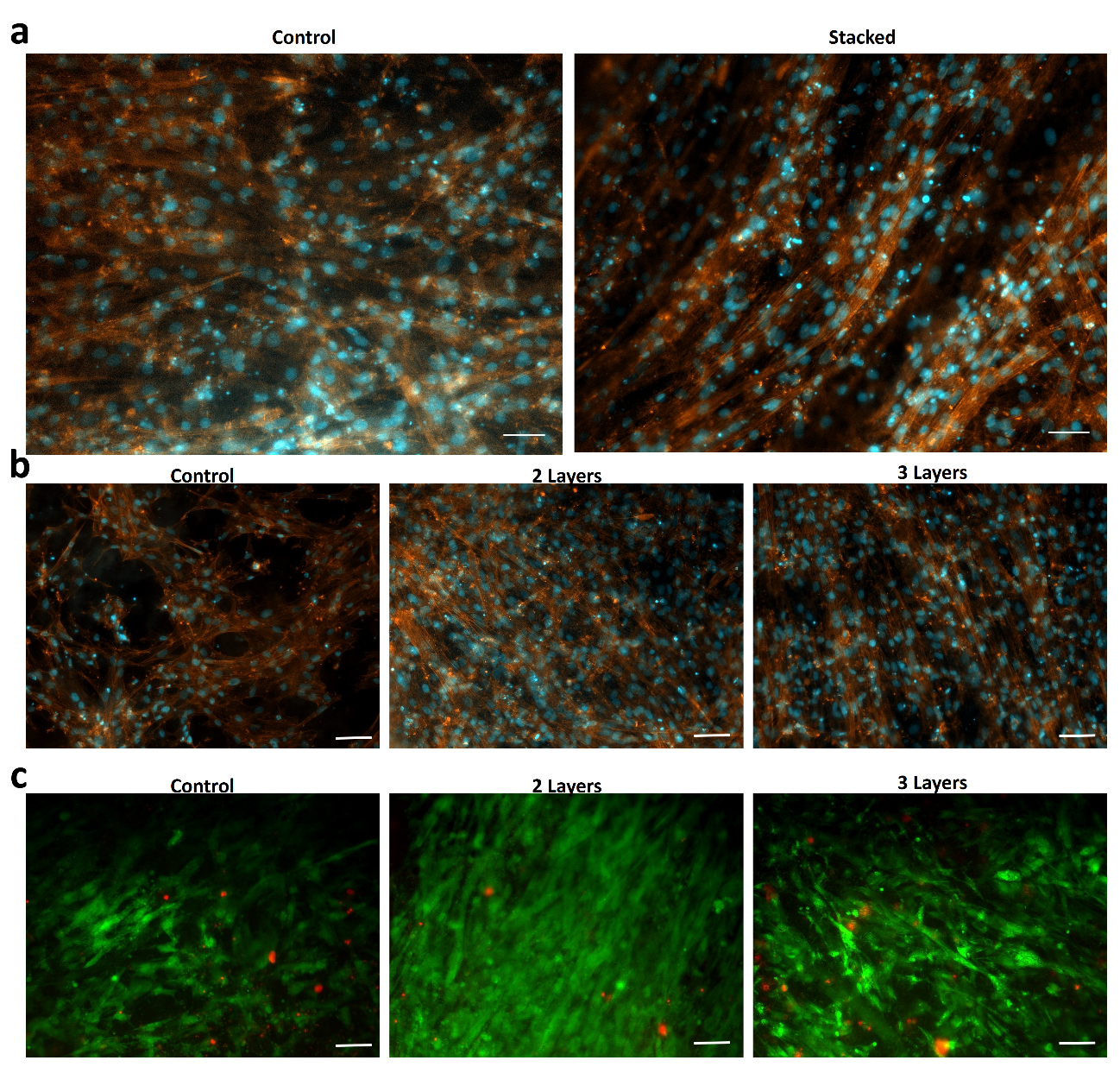


**Supplementary Figure 4.** (a) Staining of the nucleus (blue) and F-actin (orange) of H9c2 cells after 2 days of stacking in the growth medium. The left panel shows cells that were maintained on a single layer of random cellulose acetate nanofibers and the right panel those that were stacked in two layers. Scale bar = 50 µm. (b) Labeling of the nucleus (blue) and F-actin (orange) of single-layer, two-layers, and three-layer H9c2 cells stacked and grown for 6 days in a growth medium. Scale bar = 50 µm. (c) Images from the live/dead test of single-layer, two-layers, and three-layer H9c2 cells stacked and grown for 6 days in a growth medium. Live cells are shown in green and dead cells in red. Scale bar = 50 µm.


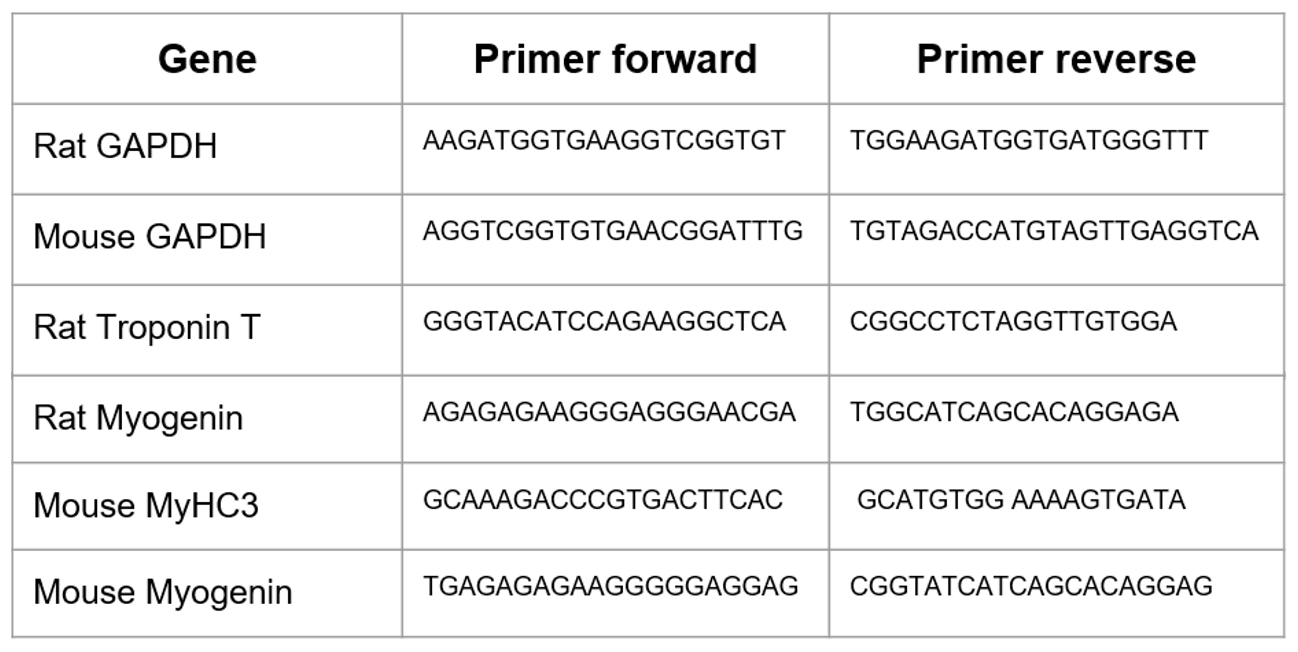


**Supplementary Table 1.** Primer sequences used in RT-PCR.
